# Study on the prognosis, immune and drug resistance of m6A-related genes in lung cancer

**DOI:** 10.1186/s12859-022-04984-5

**Published:** 2022-10-19

**Authors:** Yang Yang, Zhouyao Qian, Mingyang Feng, Weiting Liao, Qiuji Wu, Feng Wen, Qiu Li

**Affiliations:** 1 Department of Medical Oncology, Cancer Center, West China Hospital, Sichuan University, No. 37, GuoXue Xiang, Chengdu, Sichuan China; 2 West China Biomedical Big Data Center, Sichuan University, No. 37, GuoXue Xiang, Chengdu, Sichuan China; 3 Department of Gastroenterology, The First Affiliated Hospital of Nanjing Medical University, Nanjing, Jiangsu China

**Keywords:** Lung cancer, m6A, Genes, Prognosis, Immune, Drug resistance

## Abstract

**Background:**

Few studies have demonstrated that the relationship between m6A-related genes and the prognosis, tumor microenvironment and drug resistance of LC.

**Methods:**

The main results were analyzed with bioinformatics methods.

**Results:**

Hence, we found 10 m6A-related genes expressed less in tumor samples in comparison with normal ones. Using consensus clustering, all LC patients were grouped into 2 subgroups according to the overall expression of 10 differential expressed m6A-related genes. In two clusters, the OS and immune characteristics were different. We analyzed the predictive potential of 10 m6A-related genes in the prognosis of LC, and obtained a risk prognosis model on the strength of ZC3H13, CBLL1, ELAVL1 and YTHDF1 as the hub candidate genes through LASSO cox. The expression of 4 hub m6A-related genes was validated by IHC in the HPA database. The infiltration level of dendritic cell, CD4+ T cell and neutrophil that were affected by CNV level of m6A-related genes in LUAD and LUSC patients. Moreover, based on GSCALite database, we found that LUSC patients with hypermethylation tended to have a better overall survival. In terms of drug sensitivity, etoposide correlated negatively with ELAVL1, HNRNPC, RBM15B, YTHDF2 and CBLL1. ZC3H13 had positively association with afatinib, while HNRNPC was positively associated with dasatinib, erlotinib, lapatinib and TGX221. Crizotinib had a negative correlation with ELAVL1, CBLL1, HNRNPC and RBM15B.

**Conclusion:**

In conclusion, m6A-related genes are important participants in LC and the expression levels of ZC3H13, CBLL1, ELAVL1 and YTHDF1 are significant for prediction and treatment of LC. Researches of drug resistance based on m6A-related genes need to pay more attention for producing new therapeutic strategies of LC and CBLL1 may contribute to target treatment for further research.

**Supplementary Information:**

The online version contains supplementary material available at 10.1186/s12859-022-04984-5.

## Introduction

Lung cancer (LC) is the most frequent malignant tumor (11.4% of all cases) with the highest mortality (18% of the total cancer deaths) around the world [1]. Subtypes of lung cancer are mainly lung adenocarcinoma (LUAD) and lung squamous cell carcinoma (LUSC). The incidence of LUAD has increased more quickly than that of LUSC in men and especially in women over the past few decades [1, 2]. For the reason that most lung cancer patients died of lung cancer itself finally, the incidence rate and mortality rate of lung cancer tend to mirror one another [3]. Although the technologies of diagnosis and therapies such as surgical treatment, targeted treatment, radiotherapy and immunotherapy are progressing, the overall survival rate is still unfavourable [3–8]. Unluckily, only 15% of LC are discovered in early stages, which would make merits for the final prognosis, most LC patients are discovered at an advanced stage [7]. Therefore, effective biomarkers may be beneficial for early diagnosis and predicting prognosis in order to enhance the overall survival rate of lung cancer patients [9, 10].

Epigenetic regulation plays an essential role in LC. Although we have identified several layers of epigenetic regulation, including modification in the DNA or proteins levels, the mechanism of RNA modification remains unclear. N6-methyladenosine (m6A) is a modified pattern of epigenetic regulation, which takes up the most variety of mRNA modification in most eukaryotic cells. Recent researches support that almost all fields of mRNA metabolism are regulated by m6A methylation, including mRNA decay and translation in the cytoplasm, as well as pre-mRNA processing and expression in the nucleus [11–13]. Studies have suggested that dysregulation of m6A has taken a significant role of tumorigenesis and development of cancers, especially LC [14, 15]. Up to date, abnormal expression of m6A related proteins has proved to take part in some biology processes of LC, including malignant proliferation, migration, invasion, metastasis and drug resistance [16]. The further study on the methylation of m6A indicates that the prospects of early diagnosis and new treatment of LC are more and more broad.

Comprehensive analyses based on multiomics provide more information for evaluating gene expressions and functions. This study used RNA-seq downloaded from TCGA dataset to systematically analyze the expression of 21 m6A-related genes at 1039 LC patients and 107 control patients. Our aim was to evaluate the role of m6A-related genes in forecasting the prognosis of LC patients, and analyze the tumor microenvironment and drug resistance through comprehensive bioinformatics analysis (Additional file 1: Figure S1).

## Methods and materials

### Data collection and analysis

We obtained the transcriptome data, somatic mutation data and the related clinicopathological data of 1039 LC tissues and 107 normal tissues from the TCGA database (http://cancergenome.nih.gov/) as training set and GEO data as validation set. Those 21 m6A-related genes included 7 m6A writers (RBM15B, ZC3H13, KIAA1429, WTAP, METTL14, RBM15, METTL3), 2 m6A erasers (FTO and ALKBH5), 11 m6A-binding protein genes (YTHDF1, YTHDF2, YTHDF3, ELAVL1, HNRNPA2B1, HNRNPC, LRPPRC, YTHDC1, IGF2BP1, YTHDC2 and FMR1) and 1 m6A-related protein gene (CBLL1), which were received from previous literatures [17–19].

DEMGs (differentially expressed m6A-related gene) were identified between the LC samples and corresponding non-tumorous samples by the “limma” package. The “pheatmap” was utilized to draw heat maps of DEMGs.

Violin diagrams of DEMG were drawn using the “ggplot2” package. Online Gene ontology (GO) analysis was utilized to provide the definition of these proteins from three aspects, including biological process, molecular function and cellular component. Signaling pathway was analyzed at the same time. Protein–protein interaction (PPI) was achieved from the String network (http://string-db.org/cgi/input.pl). Spearman correlation coefficient with R package was performed to analyze the co-expressions among 10 DEMGs.

### Gene mutation analysis

The somatic mutation data was analyzed visually by the “maftools” R package [20]. According to RNA-seq data, coding RNAs were reserved for further analysis when their original expression count value was higher than 10 in more than three quarters of samples. The copy number variations (CNV) of m6A-related genes in LCs was analyzed in CNV module of GSCALite (http://bioinfo.life.hust.edu.cn/web/GSCALite/) [21].

### Consensus clustering of LC

According to consensus clustering, Ward’s linkage and Euclidean distance provided references to perform cluster analysis of the 10 m6A-related genes expression information in LC tissues and the corresponding clinical data. In order to infer the best K to confirm and categorize patients, the proportion of ambiguous clustering (PAC) was used as a distinct and simple unsupervised clustering method [22]. And principal component analysis (PCA) was also an intuitive way to assess the optimal k. Using “ConsensusClusterPlus” package, which consists of a total of 1,000 computation, we verified the stability and reliability of classification [23]. We used “pheatmap” package to analyze clinical correlation. The overall survival rate of different clusters was analyzed, using Kaplan Meier method.

### Construction of a gene signature and the evaluation of its prognosis and prediction

In order to evaluate the value on the prognosis of m6A-related genes and develop a potential risk model, Lasso cox regression analysis was utilized on their expression in the TCGA dataset [24, 25]. Univariable cox analysis was performed to screen out the genes that were related to survival. The minimum criterion was set as *p* value less than 0.05, then four genes and their coefficients were determined. And λ as the best penalty parameter related to the TGGA dataset were selected. The equation was used to compute the risk score of the signature [26]:

$${\text{Risk}}\;{\text{score}} = \mathop \sum \limits_{i = 1}^{n} Coefi*xi$$

In which Coefficients represents the coefficient, while xi is the representative of relative expression value of the Z-score transformation of every chosen regulator. Each patient’s risk score was calculated by this formula in TCGA dataset. In LC cases, high-risk group (the risk score of these samples exceeds 0.9539055) and low-risk group (the risk score of these samples is inferior to 0.9539055) were determined on the strength of the risk score of the tumor samples. Moreover, a nomogram was established, which assimilated the four selected genes with LC prognosis and we conducted 3-year and 5-year ROC (receiver operating characteristic curve) analysis to assess the nomogram. In addition, Cox regression analyzed the clinical characteristics correlated with the overall survival rate of LC patients with univariate and multivariate analysis, and we applied the Kaplan–Meier method to assess the practicality of risk prognostic models. Further, we conducted ROC analysis to detect the sensitivity and specificity of risk score.

### Analysis of tumor microenvironment (TME)

Tumor microenvironment contributes to tumor advances and prognosis, while immune and stromal cells are the dominant ingredients of TME [27]. About LCs, estimation of stromal and immune cells in malignant tumor tissues using ESTIMATE and XCELL algorithm was performed to cast every sample in order to deduce from the admixture of immune, stromal and other non-cancerous ingredients in the TME. We then compared the stromal score, immune score, and estimate/microenvironment score in two clusters and in the risk model and visualized with the “ggplot2” package.

### Analysis of infiltration level of immune cells and immune function

We calculated the infiltrated score of immune cells with CIBERSORT, CIBERSORT-ABS, TIMER, XCELL, QUANTISEQ, MCPCOUNTER, EPIC and ssGSEA algorithm. We also used ssGSEA and TISIDB [28] to evaluate immune functions and immunotherapy. The we got the correlation with risk model or cluster and compared the difference in the high and low risk groups. The TIMER2.0 database (http://timer.comp-genomics.org/) was performed to validate the infiltrated level of immune cells. The abundance of immune cells is estimated using a novel statistical method in the tumor microenvironment. The clinical, genomic and immunological features of tumors can be fully studied in the TIMER2.0 dataset [29]. The relationships between estimated immune infiltrates and somatic copy number alterations (sCNAs), somatic mutations, gene expression and clinical outcomes in the TCGA cohorts were allowed to investigate by the immune component consisting of four modules [30]. Hub immune-related gene mutation types were evaluated on the strength of the four modules in the TIMER2.0 dataset.

### Validation of expression of DEMGs

The Human Protein Atlas (HPA, https://www.proteinatlas.org/) website was utilized to verify the expression of proteins encoded by LUAD and LUSC selected hub genes [31], based on quantitative transcriptomics data (RNA-Seq) and spatial proteomics data achieved by tissue microarray immunohistochemical analysis. In addition, we validated the expression of DEMGs in the GEPIA 2.0 dataset (http://gepia2.cancer-pku.cn/#index).

### GSCALite

In GSCALite (http://bioinfo.life.hust.edu.cn/web/GSCALite/) [21], we established a comprehensive public resource in order to research tissue-specific gene expression and regulation by integrating the normal tissue data from the Genotype Tissue Expression (GTEx) project and the LUAD and LUSC genomics data from TCGA. The GTEx samples of nearly 1000 individuals were collected from 54 non-diseased tissue sites. We submitted all the DEMGs to the GSCALite website to analyze the methylation of the DEMGs in LUAD and LUSC based on the TCGA LUAD and LUSC samples.

Besides, we explored DEMGs related miRNA regulation network and the role of DEMGs in drug sensitivity and cancer pathway activity. In addition, this represented different expressions of DEMGs in LC subtypes. Moreover, CARE database was used to evaluate the drug resistance of these 4 selected hub genes. We defined *p* values or FDR less than 0.05 as statistically significant.

### DNA methylation

In MethSurv [32] (https://biit.cs.ut.ee/methsurv/), we determined the expression and prognostic patterns of single CpG methylation of the DEMGs in LUAD and LUSC. Beta values (ranging from 0 to 1) represent DNA methylation values. M and U are methylated and unmethylated intensity values. Every single methylation of CpG was calculated by the M/(M + U + 100) formulation.

### Statistical analysis

R software (version 4.4.30) was used to process data. The filtering conditions for all the results were: *p* < 0.05(“*”), *p* < 0.01(“**”) and *p* < 0.001(“***”).

## Results

### Gene set enrichment analysis of m6A-related genes

**Fig. 1:**
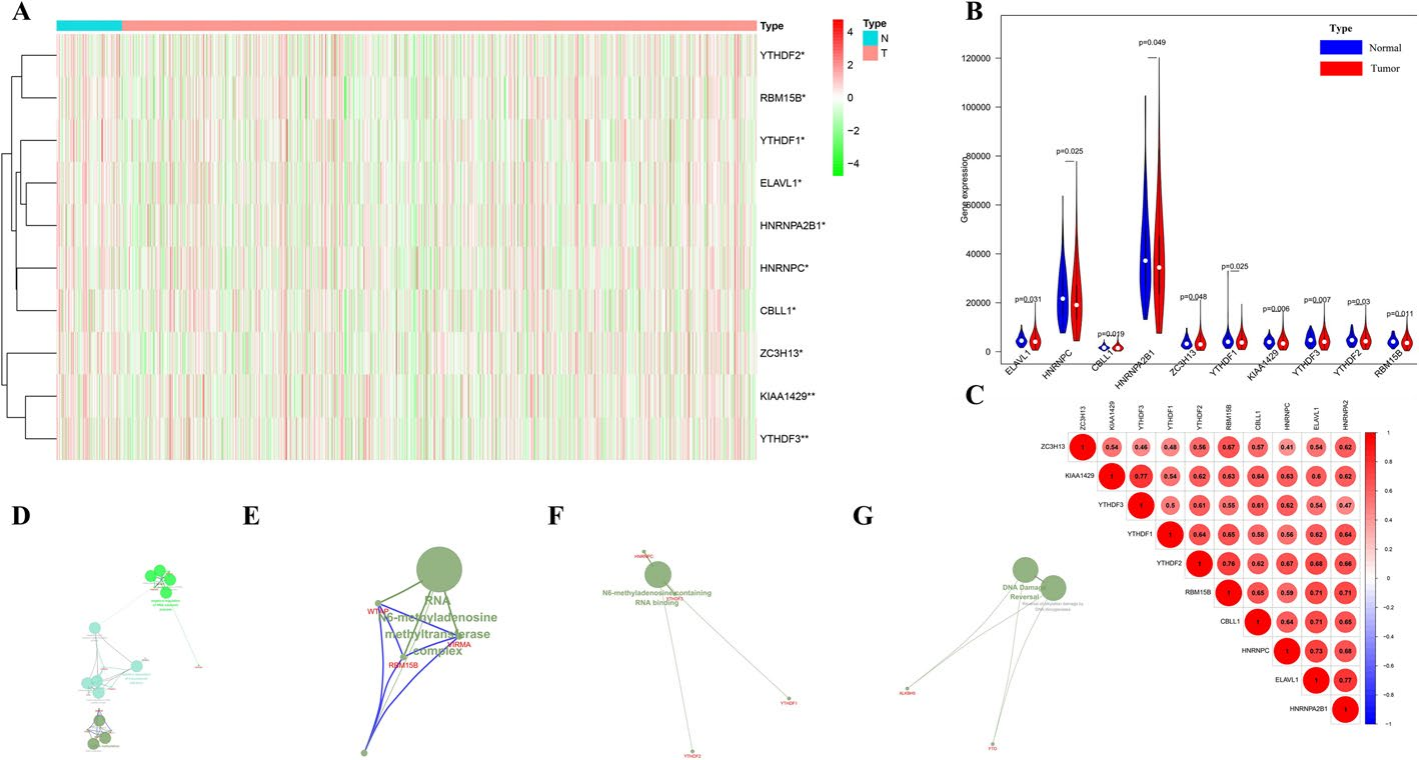
Expression of m6A-related genes and gene set enrichment analysis among them. **A** The differential-expression levels of 10 m6A-related genes in normal controls (n = 107) and LC (n = 1039) with agglomerative hierarchical clustering. **B** Vioplot diagram of 10 DEMGs in normal controls and LC. **C** Co-expressions among 10 DEMGs in lung cancer. **D–F** Biological process (BP), cellular component (CC) and molecule function (MF) of 21 m6A-related genes. **G** The pathway of 21 m6A-related genes

In view of the key function of DEMGs in tumor occurrence and development, TCGA database was used to comprehensively explore the transcription of 21 m6A-related genes. We presented the RNA levels of DEMGs as heatmaps and violin diagrams (Fig. 1A, B), which suggested that there was significant difference between the expression levels of 10 m6A-related genes in LC patients and those in normal controls. Interestingly, on the strength of the expression pattern, those 10 DEMGs (including ELAVL1, HNRNPC, CBLL1, HNRNPA2B1, ZC3H13, YTHDF1, KIAA1429, YTHDF3, YTHDF2 and RBM15B) all expressed less in tumor samples than in normal samples (Fig. 1B).

Through GO analysis, in biological process, KIAA1429 (VIRMA), RBM15B, WTAP and CBLL1 may relate to mRNA methylation; YTHDF1, YTHDF2, YTHDF3 could have the relationship with positive regulation of translational initiation; while HNRNPC, IGF2BP1, ELAVL1 and LRPPRC were likely to have the process of negative regulation of RNA catabolism. In cellular component, WTAP, KIAA1429 and RBM15B could belong to RNA N6-methyladenosine methyltransferase complex. In molecular function, HNRNPC and YTHDF3 had strong relationship with N6-methyladenosine-containing RNA binding, while the relationships among YTHDF1, YTHDF2 were weak (Fig. 1D–F). ALKBH5 and FTO were identified with the pathway of DNA Damage Reversal and reversal of alkylation damage by DNA dioxygenases (Fig. 1G).

To better investigate the interactions among the 21 m6A-related genes, co-expression analysis (Fig. 1C) was applied to explore the correlations among the 10 differential-expression genes. Then, we identified highly correlated m6A regulator gene pairs as |correlation coefficient|≥ 0.7, *p* < 0.05, which included KIAA1429 and YTHDF3, YTHDF2 and RBM15B, RBM15B and ELAVL1, RBM15B and HNRNPA2B1, CBLL1 and ELAVL1, HNRNPC and ELAVL1, ELAVL1 and HNRNPA2B1.

### Landscape of the LC mutation profiles

In the waterfall plot, the mutations of TP53, TTN, MUC16 were the top three mutated genes in LC samples, and fraction of found TP53 mutations was higher than 60% in LC samples (Additional file 2: Figure S2A and 2B). In addition, the most common type of mutation was missense mutations, the proportion of single nucleotide polymorphisms (SNPs) in the variant type was higher than that of inserted mutation or deleted mutation, and the most familiar single nucleotide variant (SNV) was C > A in LCs (Additional file 2: Figure S2B–D). Moreover, we figured up the variance of each sample and set up box plots with different colors to display the mutation types for LCs (Additional file 2: Figure S2B). Additional file 2: Figure S2F visualized the exclusive associations and co-occurrence between mutated genes. As shown in Additional file 2: Figure S2E, RTK-RAS, TP53, HIPPO were the top three pathways affected in LC sample, while TGF-Beta, MYC, Cell Cycle were the least three pathways (Additional file 2: Figure S2E).

Then, the somatic mutation profiles of 10 DEMGs were analyzed in 1059 LC patients and the altered 142 samples of SNV were selected for further analysis in the VCF format. In the oncoplot, ZC3H13, KIAA1429, CBLL1 were the top three mutant m6A genes in LC samples (Additional file 2: Figure S2G–I and Additional file 3: Figure S3C). Furthermore, the most common mutation classification was missense mutations, single nucleotide polymorphisms (SNPs) account for a higher proportion of variant types than insertion or deletion, and the most common single nucleotide variant (SNV) was C > T in the m6A RNA methylation regulators altered of LCs (Additional file 2: Figure S2H). In addition, we calculated the number of variants in each sample, and used box plots to show the mutation types for 142 samples (Additional file 2: Figure S2H).

Next, we studied the CNV of m6A-related genes in LCs. As shown in Additional file 2: Figure S2J, YTHDF1 had the highest correlation between CNV and mRNA while RBM15B had the least (Additional file 2: Figure S2J). A homozygous variation of YTHDF1 was the majority of the homozygous amplification without deletion by CNV% analysis. The probability of heterozygous amplification was relatively high in gene YTHDF1/3, KIAA1429, HNRNPA2B1 and CBLL1 (Additional file 2: Figure S2K–M).

### Consensus clustering based on the expression of 10 DEMGs

**Fig. 2:**
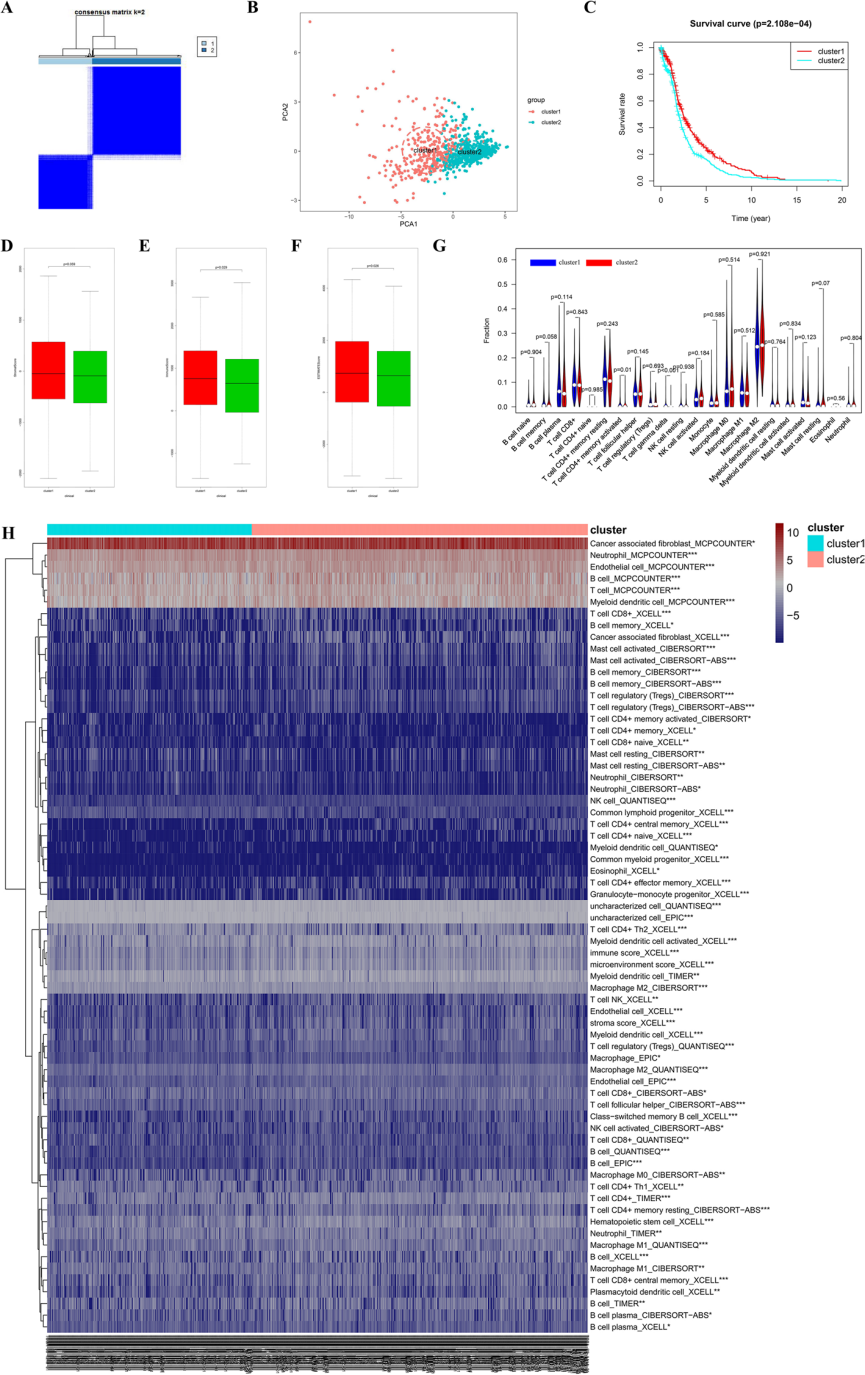
PC analysis, overall survival rate and analysis of TME of LC in 2 robust clusters. **A** The optimal cluster number was two using the ConsensusClusterPlus package. **B** Principal component analysis (PCA) was also an intuitive way to assess the optimal k. **C** Overall survival analysis between cluster 1 and cluster 2 of LC. **D–F** Comparison of stromal score, immune score and estimate score between two clusters in the TME of LC samples. **G** Violin diagram of the proportion of 22 types of immune cells involved in two clusters in LC samples. **H** The infiltration of immune cells involved in cluster1 and cluster2 in LC samples with 7 algorithm. *p < 0.05; **p < 0.01; ***p < 0.001

From Additional file 3: Figure S3A–B, the area under the cumulative distribution function (CDF) curve stabilized when k equaled to 2 (Fig. 2A, Additional file 3: Figure S3A–B). The consensus matrix shown in Fig. 2A represented the consensus for k = 2 and well-defined 2-block structure, where there was no crossover. The PC analysis showed a relatively stable partitioning of the samples in two clusters (Fig. 2B). Moreover, the prognostic analysis demonstrated significantly difference between cluster 1 and 2, and patients from cluster 1 had a better overall survival than that in cluster 2 (Fig. 2C). Then, we compared the TME in two clusters. Cluster 1 had higher immune score and estimate score than cluster 2, while there was little statistical significance in stromal score between them (Fig. 2D–F). In the violin plot (Fig. 2G), remarkable difference was found in activated memory CD4+ T cell, γδ T cell in two LC sample clusters. Therefore, we found a novel clustering for prognosis and immune characteristics of LC patients. The infiltration difference of immune cells in two clusters was shown in Fig. 2H. Among them, the infiltrated level of common lymphoid progenitor_XCELL, T cell CD4+ Th2_XCELL, Macrophage M2_CIBERSORT, T cell CD4+ Th1_XCELL, Mast cell resting_CIBERSORT, T cell CD8+ naive_XCELL, Neutrophil_CIBERSORT, Macrophage M1_CIBERSORT, Mast cell resting_CIBERSORT-ABS, T cell CD8+_QUANTISEQ, T cell CD4+ memory activated_CIBERSORT, Myeloid dendritic cell_QUANTISEQ, Neutrophil_CIBERSORT-ABS was higher in cluster 2 group.

### Construction of risk prognostic signature and the evaluation of its prognosis and prediction

With the purpose of better predicting the clinical prognosis based on these m6A-related genes in patients with LC, we got the risk model. When the log(lambda) was between − 4 and − 5, the four m6A-related gene signature (ZC3H13, CBLL1, ELAVL1 and YTHDF1) of the best prognostic model was identified. The Lasso regression coefficient of the four hub DEMGs was 4.361e−05 of ZC3H13, − 3.233e−05 of CBLL1, − 6.080e−05 of ELAVL1 and 2.777e−05 of YTHDF1, respectively.

**Fig. 3:**
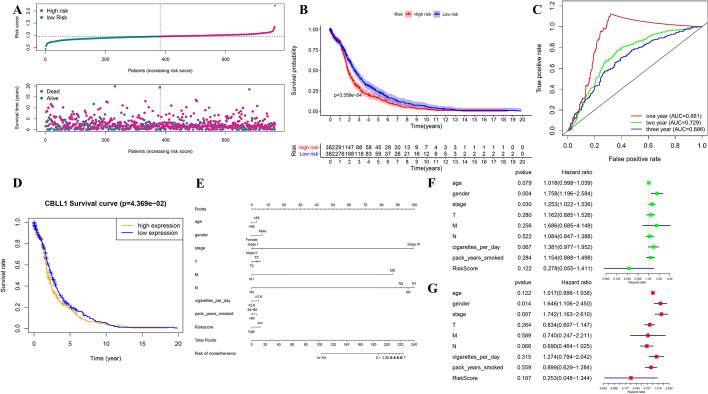
Summary of the 10 DEMGs and the selection of a 4 m6A-related gene signature in predicting LC as well as effect on LC prognosis and clinicalpathological characteristics of the 4 m6A-related gene signature. **A** Risk score and survival status for each patient in LC of TCGA datasets. **B** Kaplan–Meier OS curves for patients in the TCGA datasets designated to high- and low-risk groups depended on the risk score. **C** ROC curves demonstrated the predictive efficiency of the risk signature in LC of TCGA datasets. **D** Survival curve of CBLL1 between high- and low-expression levels in LC of TCGA datasets. **E** Nomogram for forecasting prognostic risk of LC patients. **F**, **G** Univariate and Multivariate analysis of the hazard ratios for risk score as independent prognostic elements to anticipate the overall survival

Then, risk score was calculated as the signature of LC prognosis. We divided all patients into low-risk group and high-risk group (Fig. 3A), and the OS of the two groups was carefully checked with significant difference (Fig. 3B). One-year AUC of ROC curve was 0.881 (Fig. 3C). In addition, the OS analysis represented that only low expression of CBLL1 had significantly longer survival time, whereas ELAVL1, ZC3H13 and YTHDF1 had no statistical difference (Fig. 3D). In order to better quantify the prediction results, we developed a nomogram (Fig. 3E).

Moreover, the univariate and multivariate cox analysis was respectively conducted combined with clinical characteristics. The univariate cox analysis showed that gender (HR = 1.758; *p* = 0.004) and pathologic stage (HR = 1.253; *p* = 0.030) had a significant correlation with a poor OS (Fig. 3F). The multivariate cox analysis demonstrated that gender (HR = 1.646; *p* = 0.014) and pathologic stage (HR = 1.742; *p* = 0.007) were significantly correlated to a poor OS (Fig. 3G), which was consistent with the previous analysis. In addition, GEO dataset validated the prognosis of the risk prognostic signature by the same analyses (Additional file 3: Figure S3D–G).

### Analysis of immune characteristic in the risk model

**Fig. 4:**
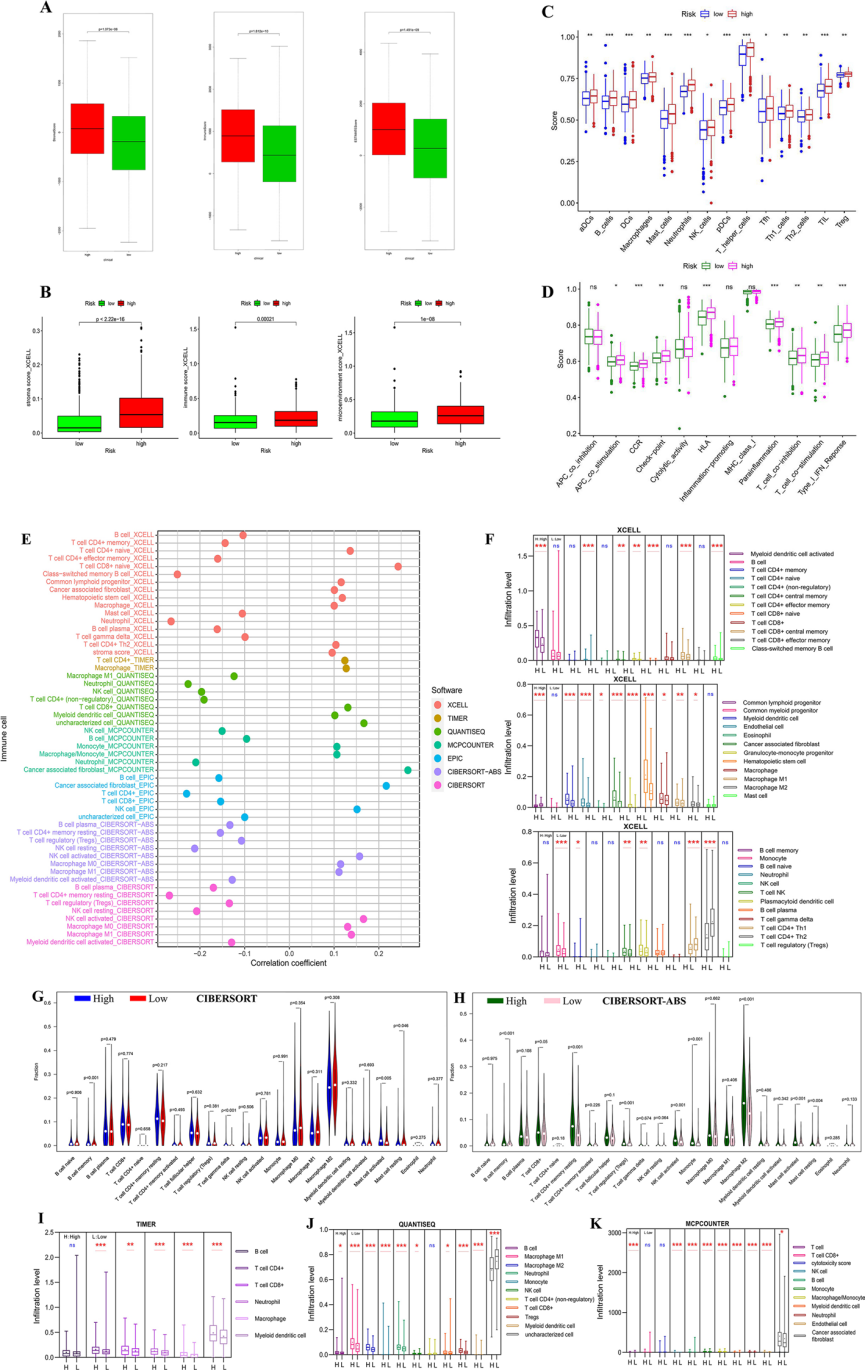
Analysis of immune characteristics of high-risk and low-risk samples. **A** Comparison of stromal score, immune score and estimate score between high-risk and low-risk groups in the TME of LC samples with CIBERSORT. **B** Comparison of stromal score, immune score and microenvironment score between high-risk and low-risk groups in the TME of LC samples with XCELL. **C** Scores of infiltration cells in high-risk and low-risk groups of LC samples with ssGSEA. **D** Scores of infiltration functions in high-risk and low-risk groups of LC samples with ssGSEA. **E** Correlation with various immune cells and risk model. **F** The infiltration of immune cells involved in high-risk and low-risk groups in LC samples with XCELL. **G** The infiltration of 22 types of immune cells involved in high-risk and low-risk groups in LC samples with CIBERSORT. **H** The infiltration of 22 types of immune cells involved in high-risk and low-risk groups in LC samples with CIBERSORT-ABS. **I** The infiltration of immune cells involved in high-risk and low-risk groups in LC with Timer. **J** The infiltration of immune cells involved in high-risk and low-risk groups in LC with QUANTISEQ. **K** The infiltration of immune cells involved in high-risk and low-risk groups in LC samples with MCPCOUNTER

We compared the immune characteristic in the risk model. There was remarkable statistical difference in immune score, stromal score and estimate score between the high- and low-risk groups, and the score of the high-risk group was higher in stromal, immune and estimate group (Fig. 4A) and XCELL groups (Fig. 4B). About the total trend of the immune cell infiltration difference, most immune cells were infiltrated more in high-risk group (Fig. 4C, E–K). About the immune functions, high risk samples got higher scores in APC co-stimulation, CCR, checkpoints, HLA, parainflammation, T cell co-inhibition, T cell co-stimulation and type I IFN response with ssGSEA (Fig. 4D).

About immune checkpoints, the expression of CD160, HAVCR2 and KDR was higher in high-risk group. The expression of CD274 was higher in low-risk group (Additional file 4: Figure S4). From TISID, the four hub DEMGs was relatively correlated with immune characteristics of LC.

### Correlation between immune cells infiltration level and hub DEMGs

We performed a correlation of m6A-related genes expressions with immune infiltration level in LUAD and LUSC. When there was no immune infiltration in LC, four hub DEMGs had no statistical significance in LUAD. Except for ZC3H13, the other three genes (CBLL1, ELAVL1 and YTHDF1) were positively correlated with purity state of tumor cell in LUSC. Intriguingly, there were no variation between YTHDF1 level and either of 6 infiltrates in both LUAD and LUSC except with CD4+ T cell and macrophage infiltrate in LUSC. CBLL1 positively related to macrophage in LUSC, CD8+ T cell, macrophage and neutrophil in LUAD and LUSC. Besides, ELAVL1 and ZC3H13 had remarkable positive correlations with almost every 6 immune infiltration cells in LUAD and LUSC. These results provided important evidence for the correlation between immune infiltrates and the expression of DEMGs in LUAD and LUSC (Additional file 5: Figure S5A–W).

We analyzed somatic copy number variation of m6A-related genes in LUAD and LUSC. The results demonstrated that an arm-level gain of CBLL1 gene reduced the immune infiltration of CD8+ T cell, dendritic cell and neutrophil in LUSC, while an arm-level deletion of CBLL1 gene attenuated neutrophil infiltrate in LUAD and an arm-level gain or a high amplication of CBLL1 increased the immune infiltration of CD8+ T cell in LUAD. Besides, an arm-level deletion of ELAVL1 attenuated the immune infiltration of dendritic cell and neutrophil in both LUAD and LUSC, as well as B cell in LUSC and CD4+ T cell in LUAD. And an arm-level gain of ELAVL1 also decreased dendritic cell and neutrophil infiltrates in LUSD. In addition, an arm-level gain of YTHDF1 and attenuated CD4+ T Cell infiltrate in LUAD, while an arm-level deletion of YTHDF1 decreased neutrophil infiltrate in LUSD. Moreover, an arm-level deletion and an arm-level gain of ZC3H13 reduced CD4+ T cell infiltrate in LUAD. Interestingly, macrophage had almost none impact by CNV of DEMGs in both LUAD and LUSC. These results demonstrated that neutrophil, CD4+ T cell and dendritic cell infiltration levels were particularly affected by CNV of DEMGs in patients with both LUAD and LUSC (Additional file 5: Figure S5AK–AY).

To further investigate the mutation of the four hub DEMGs, we found that the mutated CBLL1 attenuated the immune infiltration of neutrophil in LUSC, while the mutated ZC3H13 improved the macrophage infiltration in LUSC. In addition, there was no statistical significance in other mutated genes infiltrated by immune cells. These findings suggested that immune cells, which were affected by mutation of DEMGs made little impact in patients with both LUAD and LUSC (Additional file 5: Figure S5AI and 5AJ).

In addition, we discovered that the infiltration levels of B cell had a positive correlation with the OS of LUAD patients, while the infiltration levels of dendritic cell negatively related to the OS of LUSC patients (Additional file 5: Figure S5AA–AH).

Meanwhile, we validated the correlation of the other 6 DEMGs (HNRNPA2B1, HNRNPC, KIAA1429, RBM15B, YTHDF2 and YTHDF3) with immune cells in LUAD and LUSC. Intriguingly, HNRNPA2B1 correlated positively with almost every 6 immune infiltration cells in LUAD and LUSC, while other DEMGs had less correlations with infiltrates (Additional file 6: Figure S5BA–CI). The somatic copy number variation of 6 DEMGs was also analyzed in LUAD and LUSC, the results showed an arm-level gain of HNRNPA2B1 attenuated neutrophil infiltrate in LUSC. An arm-level gain of YTHDF3 decreased the infiltration level of dendritic cells in LUAD. The results were consistent with our previous findings that neutrophil and dendritic cell infiltration levels were particularly affected by CNV of DEMGs in patients with LC (Additional file 7: Figure S5DQ–DR). In the terms of mutation of 6 DEMGs, the mutated HNRNPA2B1 attenuated the immune infiltration of CD4+ T cells in LUAD, while the mutated YTHDF3 decreased immune infiltration of B cells in LUAD. And HNRNPC decreased CD4+ T cells and dendritic cells infiltration in LUAD. These findings demonstrated that immune cells, which were affected by mutation of DEMGs made little impact in patients with both LUAD and LUSC (Additional file 7: Figure S5DM–DP). Similar to previous findings, the infiltration levels of B cell positively related with the OS of LUAD patients, while the infiltration levels of dendritic cell had a negative relationship with the OS of LUSC patients (Additional file 7: Figure S5DA–DL).

### Relationship between immune functions and four hub DEMGs

The expression of ELAVL1 and YTHDL1 was negatively correlated to chemokine receptor, MHC molecular in LUAD and LUSC. The expression of CBLL1 was positively correlated to CCL7, CCL8, CXCL9, CXCL10, CXCL11, CCR5, TAP1, TAP2, memory B cell, active CD4+ T cell, Th2 T cell, MICB, CD274 and PDCD1LG2. The expression of ZC3H13 was positively correlated to CCR8, Tem CD4+ T cell, Th2 T cell, memory B cell, IL6R, TNFSF15, KDR and TGFBR1. The expression of ELAVL1 was positively correlated to XCL1, active CD4+ T cell, CD276, PVR, TNFSF18, ULBP1 and PVRL2. The expression of YTHDL1 was positively correlated to CXCL17, CD276, PVR and PVRL2. The remaining factors were negatively or not correlated to the four hub DEMGs (Additional file 8: Figure S6).

### Methylation and pathway

**Fig. 5:**
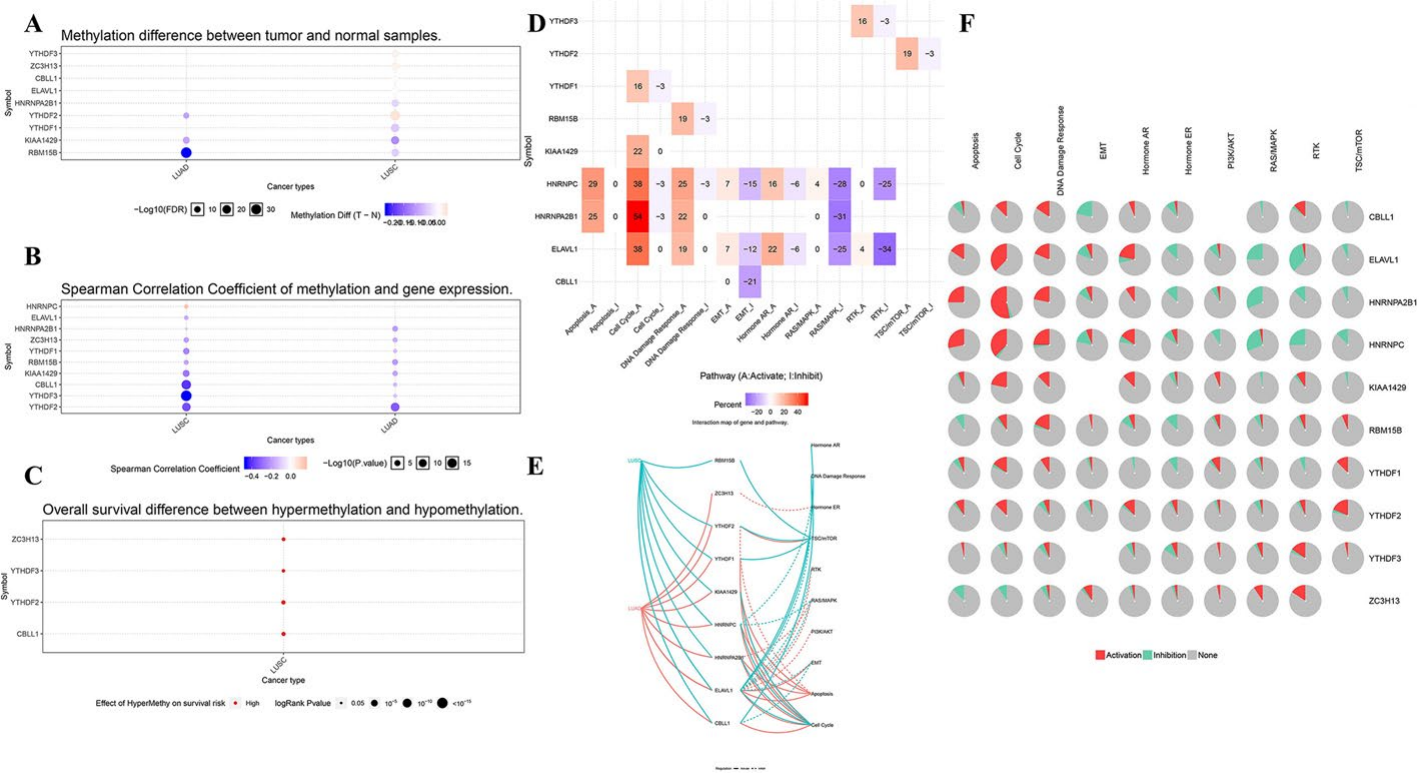
The association between m6A-related genes and methylation and the role of m6A-related genes in cancer-related pathways. **A** Methylation difference between tumor and normal samples in LUAD and LUSC. **B** Spearman Correlation Coefficient of methylation and gene expression in LUAD and LUSC. **C** Overall survival difference between hypermethylation and hypomethylation in LUSC. **D** Global percentage of activity of m6A-related gene pathway in LUAD and LUSC. A represents Activate, while I represents Inhibit. **E** Interaction map of m6A-related genes and pathway in LUAD and LUSC. Solid line represents activation, while dotted line means inhibition. **F** Pie percentage of activity of m6A-related gene pathway in LUAD and LUSC. Red part means activation, while green part represents inhibition

We collected expression profiles of the m6A-related genes with methylation in order to assess the potential effects of disrupting m6A-related genes in LUAD and LUSC patients. These results suggested that YTHDF2, KIAA1429 and RBM15B were remarkably down-regulated in LUAD compared with normal samples. Besides, significantly high levels of YTHDF3, ZC3H13, CBLL1, ELAVL1 and YTHDF2 in LUSC compared to normal tissues, while HNRNPA2B1, YTHDF1, KIAA1429 and RBM15B were downregulated in LUSC in comparison with normal tissues (Fig. 5A). In addition, methylation led to down regulation of HNRNPA2B1, ZC3H13, YTHDF1, RBM15B, KIAA1429, CBLL1, YTHDF3, YTHDF2 in LUAD and LUSC tissues, and of ELAVL1 in only LUSC tissues, while mediate HNRNPC upregulation in LUSC tissues (Fig. 5B). Furthermore, LUSC patients with hypermethylation tended to have a better overall survival (Fig. 5C). To further confirm and validate our findings, DNA methylation of all DEMGs were analyzed. We presented heatmap and prognostic value of DNA methylation expression levels of DEMGs in LUAD and LUSC (Additional file 9: Figure S7 and Additional file 11: Table S1). DNA methylation expression levels concluded that cg01043729 from ELAVL1 and cg06720244 from ZC3H13 had the highest DNA methylation levels and significant prognostic value (likelihood ratio (LR) test *p* value < 0.05) in LUAD.

Moreover, we made an exploration of DEMGs in cancer hallmark pathways. The results indicated that hormone AR, DNA damage response, TSC/mTOR and cell cycle were activated in LUSC, TSC/mTOR, apoptosis and cell cycle were activated in LUAD and hormone ER, RTK, RAS/MAPK, PI3K/AKT and EMT were inhibited in both LUSC and LUAD (Fig. 5D–F).

### Drug sensitivity and DEMGs

**Fig. 6:**
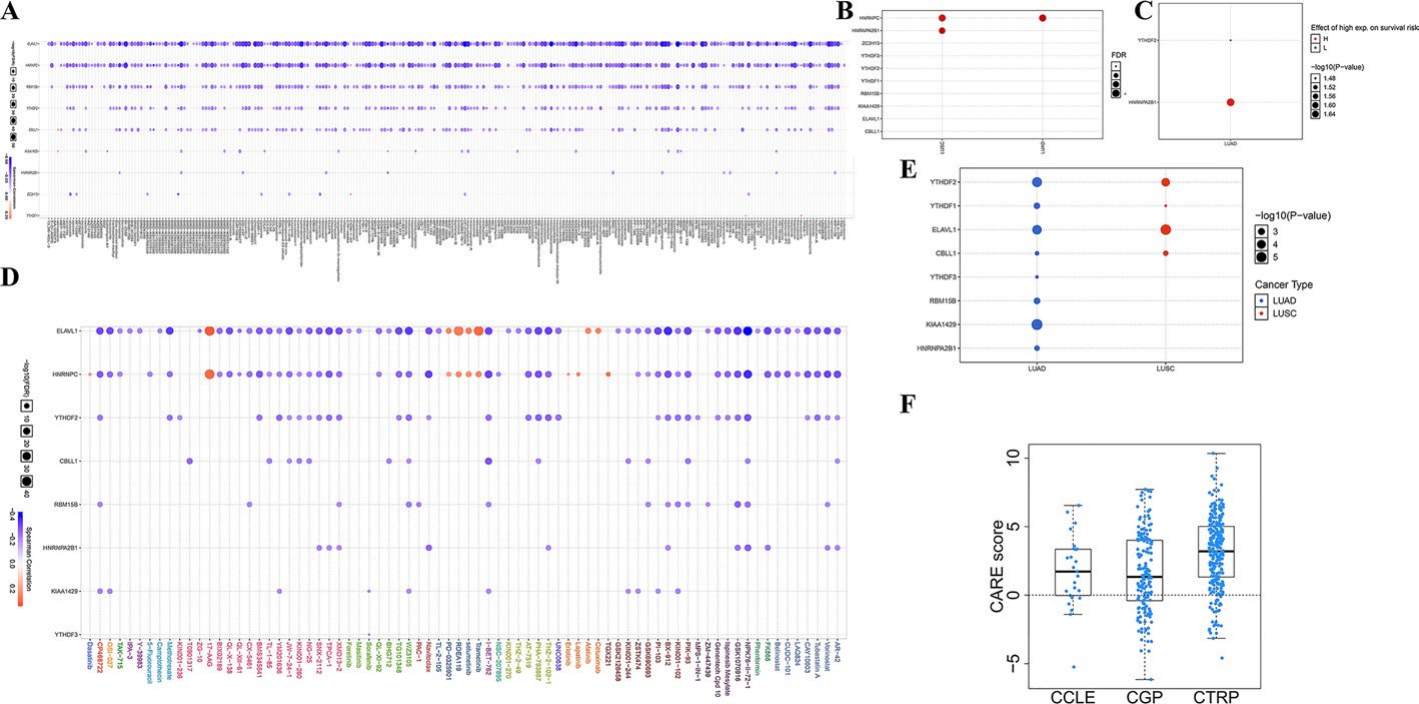
Gene expression, correlation between m6A-related genes and drug resistance in LUAD and LUSC. **A** Correlation between m6A-related genes and drug sensitivity in CTRP database. *p* value or FDR < 0.05 was considered as significant. **B** FDR of 10 DEMGs in LUAD and LUSC. **C** Effect of high expression on survival risk of 10 DEMGs in LUAD. **D** Correlation between m6A-related genes and drug sensitivity in GDSC database. *p* value or FDR < 0.05 was considered as significant. **E** Differential expression of 10 DEMGs in LC subtypes. **F** CARE score of CBLL1 in CCLE, CGP and CTRP database

The analysis of gene set drug resistance was obtained from GDSC/CTRP IC50 drug data. The spearman correlation is representative of correlation between the gene expression and the drug. The negative correlation represents that the gene low expression is sensitive to the drug. In CTRP, low level of almost all the DEMGs, including ELAVL1, HNRNPC, RBM15B, YTHDF2, CBLL1, KIAA1429, HNRNPA2B1 and ZC3H13 were negatively associated with drugs. CBLL1 and KIAA1429 had positive correlation with abiraterone, ZC3H13 and HNRNPC positively correlated with FGIN-1-27 and YTHDF1 was positively associated with tozasertib. Paclitaxel and docetaxel had negative correlations with ELAVL1, HNRNPC and RBM15B, while etoposide correlated negatively with ELAVL1, HNRNPC, RBM15B, YTHDF2 and CBLL1 (Fig. 6A). In GDSC, ELAVL1 and HNRNPC had both positive and negative correlation with specific drugs, while RBM15B, YTHDF2, CBLL1, KIAA1429, HNRNPA2B1 and ZC3H13 were negatively correlated with the drugs. 17-AAG, PD-0325901, RDEA119, selumetinib, trametinib were positively correlated with both ZC3H13 and HNRNPC. ZC3H13 had positively association with afatinib and cetuximab, while HNRNPC was positively associated with dasatinib, erlotinib, lapatinib and TGX221. Crizotinib had a negative correlation with ELAVL1, CBLL1, HNRNPC and RBM15B (Fig. 6D). In addition, the false discovery rate of HNRNPC was positive in both LUAD and LUSC, while the false discovery rate of HNRNPA2B1 was positive in LUSC (Fig. 6B). Moreover, higher level of HNRNPA2B1 and lower level of YTHDF2 in LUSC had a better overall survival (Fig. 6C). As shown in Fig. 6E, YTHDF1/2, ELAVL1 and CBLL1 expressed in LUAD and LUSC, and the expression level of YTHDF2, ELAVL1 was extremely high. YTHDF3, RBM15B, KIAA1429 and HNRNPA2BA expressed only in LUAD (Fig. 6E). Moreover, the CARE score of only CBLL1 was higher than 0 in CCLE, CGP and CTRP in the four hub DEMGs (Fig. 6F).

### Validation of expression of DEMGs

**Fig. 7:**
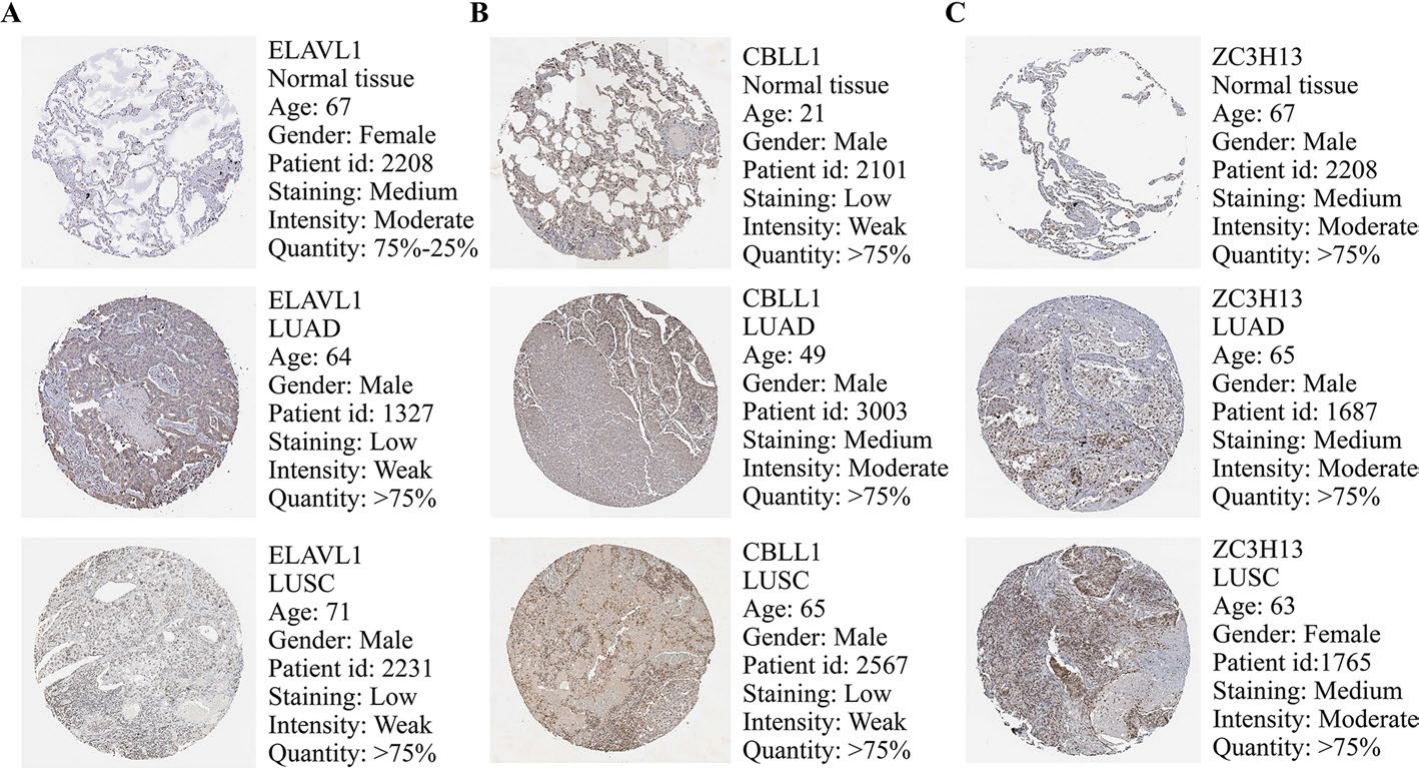
Expression of 4 selected hub DEMGs in LUAD and LUSC samples. **A**–**C** The immunohistochemical data were obtained from the Human Protein Atlas (HPA). Except for YTHDF1, expression profiles of the ELAVL1, CBLL1 and ZC3H13 in normal, LUAD and LUSC clinical samples are shown

The expression levels of proteins which were encoded by the 4 selected central genes correlated with LUAD and LUSC were obtained from the HPA website. The HPA website reports no data on proteins encoded by YTHDF1, while Fig. 7 shows the expression profiles of the other 3 genes in LUAD and LUSC clinical samples. The protein level of ELAVL1 was downregulated in LUAD and LUSC tissues in comparison with normal tissues, while the protein expression of CBLL1 was upregulated in LUAD in comparison with normal tissues. CBLL1 overexpressed in both LUSC tissues and normal tissues, while ZC3H13 overexpressed in LUAD, LUSC and normal tissues (Fig. 7A–C). And the overall survival analysis of HPA suggested that low expression of CBLL1 had a strong relationship with a poor prognosis in LC patients (Additional file 10: Figure S8A), but the other three genes had no statistical significant correlation. Then, we utilized GEPIA website to validate the expression of these 4 selected hub genes. The results demonstrated that CBLL1, ELAVL1 and YTHDF1 expressed more in tumor samples than in normal samples of LUAD and LUSC patients, while the expression level of ZC3H13 in tumor samples is lower than in normal samples of LUAD and LUSC patients (Additional file 10: Figure S8B–C).

## Discussion

In our study, we aimed to analyze the prognosis, tumor microenvironment and drug resistance of m6A-related genes in LC. We developed a risk prognostic signature consisting of ZC3H13, CBLL1, ELAVL1 and YTHDF1 in 1039 LC patients from TCGA dataset as the training set by Lasso cox analysis and GEO dataset as the verified set. Through OS analysis and ROC analysis, we validated the sensitivity and specificity of this gene signature.

YTHDF1 was recognized as ‘m6A reader’ and could identify m6A marks and mediate m6A functions. The expression levels of YTHDF1 had a tight correlation with cancers proved by many researches, such as LC, hepatocellular carcinoma (HCC), and colorectal cancer (CRC) [33, 34]. Studies [35] have discovered that YTHDF1 could regulate durable neoantigen-specific immunity, which suggested that YTHDF1 may acted as a potentially therapeutic target in the aspect of immunotherapy. Shi et al. [34] found that YTHDF1 expressed lower in normal samples than in tumor ones. The knockdown of YTHDF1 inhibited cell proliferation of NSCLC by managing the translational efficiency of cyclin D1 (CCND1), cyclin-dependent kinase 2 (CDK2) and cyclin-dependent kinase 4 (CDK4). Their studies also suggested the abilities of YTHDF1 included regulating cell responses to cisplatin-dependent chemotherapy, which impacted the treatment and prognosis. Jin et al. [36] found that YTHDF1 accelerated YAP translation and then enhanced the growth, invasion, and EMT of NSCLC cells. Sheng et al. [37] found that YTHDF1 took part in maintaining LC cell metabolism and development. Studies about YTHDF1 and LC are rather abundant, while further researches on mechanism of YTHDF1 and LCs need to be considered.

ZC3H13, a zinc finger protein, was recognized as ‘m6A writer’ and could install m6A methylation transcriptionally on RNA. Previous studies have discovered that ZC3H13 had a somatic frame-shift mutation in CRC. In Zhu’s study [38], the results suggested that ZC3H13 may play the role of regulating upstream of RAS-ERK signaling pathway, which led to inhibit cell proliferation and invasion in CRC. Moreover, they found that the decreasing level of ZC3H13 was correlated with advanced TNM stage, positive regional lymph node metastasis. Gong et al. [39] suggested that ZC3H13 expressed less in breast cancer cell patients, and when METTL4 and ZC3H13 expressed particularly low, the prognosis tended to be unfavorable in four breast cancer subtypes through survival outcome analysis. In addition, their down-regulation had a correlation with tumor progression of triple-negative breast cancer patients. ZC3H13 and METTL14 were strongly related to APC (an antagonist of the Wnt signaling pathway), meaning that ZC3H13 and METTL14 are involved in the regulation of invasion, proliferation, and metastasis of tumor cells. Immune infiltration analysis indicated that METTL14 and ZC3H13 could facilitate breast cancer invasion by influencing immunosuppression-related pathways. Studies about ZC3H13 were limited in the researches of insects, colorectal cancer and breast cancer. However, there was no studies involving in ZC3H13 and LC, which needed further experiments and analyses.

CBLL1 was recognized as one of m6A-related genes. Hui et al. [40] found that CBLL1 expressed lower in adjacent non-tumor tissues than in NSCLC tissues. Previous studies have discovered that CBLL1 could accelerate the proliferation and invasion of A549 and H460 cells. And CBLL1 promoted G1/S cell cycle transition, resulting in the proliferation of NSCLC cells. In terms of lncRNA regulation, through miR-212-3p/CBLL1 axis, the knockdown of XIST suppressed the proliferation, migration, invasion and EMT of NSCLC cells [41]. In conclusion, CBLL1 may be used as a tumorigenic marker in the progression of NSCLC. Further studies may pay more attention to other biological functions of CBLL1 and potential target treatment of CBLL1 in LC patients.

ELAVL1/HuR (embryonic lethal abnormal vision like 1/human antigen R) was also recognized as ‘m6A reader’ and participated in cell differentiation and stress response [42]. ELAVL1 could promote the proliferation of tumor cells by directly binding ER or regulating epidermal growth factor receptor-2 (ERBB2), cyclooxygenase-2 (COX-2) and VEGF-A through a series of signal transduction pathways [43]. Overexpression of snail downregulated cadherin expression through Snail-ETV7-SERPINE1 pathway, promoting EMT and enhancing tumor cell invasion and metastasis [44]. In addition, ELAVL1 protein recognized and bound to Scribble mRNA 3'-UTR, increasing the transcription level of scribble. As an agonist of p38 MAPK pathway, scribble promoted ELAVL1 nuclear shuttle, indirectly promoting Snail transcription level, and accelerating EMT process [45]. In conclusion, studies about ELAVL1 have involved the mechanism and functions in tumors. However, few studies about ELAVL1 and LC have been explored and other ELAVL family protein have not been investigated about their mechanism and cell functions. Hence, to solve these problems, further experiments and analyses were needed.

Here, we have to recognize that single m6A-related gene does not make sense about predicting the prognosis of LC because cancer is complicated [46]. The hub-gene signature was established to evaluate the cancer survival situation, which benefited patient prognostic prediction, drug resistance and treatment. In our study, the four DEMGs-signature (ZC3H13, CBLL1, ELAVL1 and YTHDF1) was validated to have significant relationship with clinical characteristics by univariate and multivariate cox analysis, which suggested their potential usage of forecasting the prognosis of LC. In addition, TME and drug resistance of the four DEMGs-signature were analyzed, which demonstrated the clinical value of novel immunotherapy and solutions of drug resistance in chemotherapy of LC.

Our results indicated that the score of high-risk model was higher in stromal, immune and estimate than low-risk model, which was consistent with the overall survival. B cell memory, γδ T cell and activated mast cell infiltrated more in low-risk groups. T cell gamma delta can kill target cells by directly recognizing protein antigens [47], and can also mediate killing through TCR and NKG2D non-specifically, which gives itself the function of protective immune surveillance [48], leading to anti-tumor immunity. B cell memory could promote more mature B cells, resulting in the ability of tumor immunology [49]. The mechanism of mast cell activated is still elusive in tumor immunology. Therefore, we suggested that the infiltration of three immune cells improved the ability of tumor immunology, as well as attenuated the incidence of LC.

We also found that ELAVL1 and ZC3H13 had positive correlations with almost 6 infiltrate cells in LUAD and LUSC, while YTHDF1 correlated positively with CD4+ T cell and macrophage cells in LUSC. CBLL1 was positively associated with macrophage in LUSC, and CD8+ T cell, macrophage and neutrophil in LUAD. This may suggest that ELAVL1 and ZC3H13 have a better anti-tumor immunity, which was in accordance with previous studies [39, 44]. In DNA methylation analysis, our study found that the prognostic value of ELAVL1 and ZC3H13 in a single CpG were significant in LUAD development. We found prognostic significance of DNA methylation expression levels in cg01043729 from ELAVL1 and cg06720244 from ZC3H13. In our study, neutrophil, CD4+ T cell and dendritic cells were significantly affected by the CNV of DEMGs in both LUAD and LUSC, which proved that neutrophil, dendritic cell and CD4+ T cell were more sensitive in the immune microenvironment of LUAD and LUSC, in keeping with other researches [50]. Besides, our results suggested that mutation of m6A-related genes had little correlation with immune cells in both LUAD and LUSC patients, which may because of the limitation of samples. Besides, we validated the correlation of the other 6 DEMGs with immune cells, and found only HNRNPA2B1 had a positive correlation with almost every 6 immune infiltration cells in LUAD and LUSC, which may suggest a better anti-tumor immunity.

Moreover, in our study, the infiltration levels of B Cell had a positive correlation with the overall survival of LUAD patients, while the infiltration levels of DCs were negatively related to the overall survival of LUSC patients. Previous study [49] indicated that the infiltration of B Cell and the formation of tertiary lymphoid structures were positively correlated with the response to immunotherapy, which is in keeping with our findings. Dendritic cell is the most powerful APC (Antigen presenting cell), which could stimulate the proliferation of naive T cell, leading to kill tumors, and have a positive correlation with overall survival [50]. However, our finding was opposite to previous studies, which may due to individual differences. We analyzed the tumor immune microenvironment, correlation and immune infiltration levels based on the 4 DEMGs. Because of the limitation of statistics, more researches and experiments need to be conducted to analyze the TME in a large cohort study, and explore immunotherapy of LC based on the 4 DEMGs.

Current first-line and second-line chemotherapy drugs include platinum drugs (carboplatin and cisplatin), paclitaxel, docetaxel, pemetrexed, gemcitabine, vinorelbine, etoposide and so on. Targeted Drugs include Tyrosine kinase inhibitors (TKI) (such as crizotinib, afatinib, erlotinib, trametinib, brigatinib and gefitinib), VEGF or VEGFR inhibitors (such as bevacizumab and ramuciruma), cetuximab and so on. Drug resistance is a key factor leading to the failure of tumor treatment. Therefore, studies in-depth of the molecular mechanism of drug resistance could provide basis theoretically for guiding potential drugs and overcoming drug resistance. The m6A-related genes also have a strong connection with drug resistance in LC.

Our findings suggested that paclitaxel and docetaxel had negative correlations with ELAVL1, HNRNPC and RBM15B, which meant paclitaxel and docetaxel were sensitive to ELAVL1, HNRNPC and RBM15B in LC. MONZO extracted DNA from the tissues of 43 patients with NSCLC who received paclitaxel chemotherapy, and found that 33% of the patients had mutations in the β-microfilament gene and its effective rate of chemotherapy is 0, which indicated that mutation of tubulin related to drug resistance [51]. For the reason that paclitaxel-induced apoptosis depends on Raf-1/Bcl-2, P28, caspases-3 and other important apoptotic genes, defects of these genes and pathways will decrease the effectiveness of paclitaxel and docetaxel chemotherapy in LC patients [52, 53]. Therefore, new paclitaxel and docetaxel chemotherapy based on ELAVL1, HNRNPC and RBM15B should be considered to attenuate drug resistance, resulting in enhancing the effectiveness of LC chemotherapy.

Etoposide was negatively correlated with ELAVL1, HNRNPC, RBM15B, YTHDF2 and CBLL1, which suggested that etoposide was sensitive to ELAVL1, HNRNPC, RBM15B, YTHDF2 and CBLL1 in LC. Etoposide, a Topoisomerase inhibitor, is the basis of chemotherapy to pulmonary neuroendocrine tumor [54]. Though the initial efficacy is well, the effective period is short and almost after 4–6 treatment cycles, drug resistance appears, tumors grow rapidly and begin metastasis and 2-year survival rate is less than 10%. Therefore, reversing or decreasing drug resistance of etoposide becomes the most important part in curing pulmonary neuroendocrine tumor [55]. ELAVL1, HNRNPC, RBM15B, YTHDF2 and CBLL1 may be potential targets for etoposide to decrease drug resistance and prolong the action time to cure LC.

Crizotinib was negatively associated with ELAVL1, CBLL1, HNRNPC and RBM15B, which proved that crizotinib was sensitive to ELAVL1, CBLL1, HNRNPC and RBM15B.With the fact that crizotinib is a kinase inhibitor targeting c-MET/ALK/ROS1, which is the fisrt-line chemical treatment to NSCLC with ALK mutations, most NSCLCs are resistant to crizotinib treatment without considering the overexpression of c-MET in about 35–72% NSCLC. In order to improve the sensitivity of criztinib treatment in NSCLCs, chidamide would downregulate the expression of mRNA m6A methylation regulators WTAP and METTL3, which decreased the expression of c-MET, resulting that crizotinib could sensitize more of NSCLC cells in a c-MET/HGF-dependent manner [56]. Based on these findings, more experiments and researches need to be done to study whether crizotinib based on ELAVL1, CBLL1, HNRNPC and RBM15B sensitize more of NSCLC cells, thus attenuating drug resistance in LC.

Afatinib had a positive correlation with ELAVL1, which suggested that afatinib was resistant to ELAVL1 in LC. Recently, it was [57] deeply analyzed the relationship between m6A methylation and Alfatinib resistance in NSCLC. They took sensitive cell lines of afatinib as controls, and found that more genes were modified by m6A in afatinib resistant groups, which resulted that there were changes in the overall gene expression profile. They speculated that genes modified by m6A methylation disrupted the normal cell cycle, leading to the development of afatinib resistance in NSCLC, based on the finding that differential expression genes were largely enriched in cell cycle through gene function analysis. Therefore, genes modified by m6A methylation, especially by ELAVL1 tend to improve afatinib resistance and have a poor efficacy in NSCLC.

Erlotinib correlated positively with HNRNPC, which proved that erlotinib was resistant to HNRNPC. Previous studies indicated that the resistance of erlotinib to NSCLC was mediated by two mechanisms [58–60]. One is the changes of EGFR conformation by acquiring secondary mutations, thus improving the resistance to erlotinib. The other is an alternative mechanism of activating development and proliferation, which includes activation oncoproteins such as MET, HER2, BRAF or PIK3CA, cellular transformation induction including epithelial to mesenchymal transition (EMT) or transformation from NSCLC to small cell lung cancer (SCLC). And some mechanisms of erlotinib resistance to NSCLC are still unknown [61]. Future studies may focus on the internal mechanism of m6A-related genes, especially HNRNPC and erlotinib, thus conducting a new erlotinib therapeutic strategy based on HNRNPC to prevent drug resistance in LC.

Trametinib had a positive association with ELAVL1 and HNRNPC, which suggested that Trametinib was resistant to ELAVL1 and HNRNPC. A review [62] about trametinib proved that few basic experiments and clinical trials had been conducted to analyze trametinib resistance to LC. Therefore, researches based on ELAVL1 and HNRNPC and related analysis of trametinib resistance need to be done for providing a new therapeutic choice to decrease drug resistance in LC.

Cetuximab was associated with ELAVL1 positively, which proved that cetuximab was resistant to ELAVL1. Clinical trials [63] have shown that cetuximab could produce drug resistance in the treatment of NSCLC, which may be related to the disorder of multiple transmission pathways, including the activation of alternative signaling pathways, receptor mutations, ligand autocrine/paracrine production, and the constitutive activation of downstream signaling proteins. Researches of cetuximab resistance based on ELAVL1 may pay more attention to find out internal mechanisms of drug resistance and produce a new cetuximab therapy to increase the effectiveness of LC treatment.

Our study discovered that the 10 m6A-related genes expressed differently in LUAD and LUSC. Considering about the false discovering rate and the expression survival analysis, HNRNPC and HNRNPA2B1 also need to pay more attention and may be used as a target gene about the OS in LUAD and LUSC. Due to the functions of the 4 m6A-related gene signature described above, we assessed the drug resistance of these 4 hub genes in CARE dataset, and ultimately found that the CARE scores of CBLL1 were higher than 0 in CCLE, CGP and CTRP, which indicated that CBLL1 may be a potential prognostic target for reversing tumor progression in LC. Current studies have shown that there may be a tight correlation between m6A-related genes and drug sensitivity in LUAD and LUSC, but the specific mechanism is still elusive. Studying the correlation and interaction between m6A-related genes and tumor drug resistance-related genes may be a new direction for future research on tumor drug resistance mechanisms in LUAD and LUSC.

The advantage of our study is that it is the first time to analyze the prognosis, tumor microenvironment and drug resistance of m6A-related genes in LUAD and LUSC by performing integrated bioinformatics analyses. Second, we successfully established a risk prognostic signature consisting of 4 hub m6A-related genes (ZC3H13, CBLL1, ELAVL1 and YTHDF1) and verified the sensitivity and specificity of the gene signature in LC. Third, we made an analysis of the tumor immune microenvironment, correlation and immune infiltration levels based on the 4 DEMGs, which make benefits for immunotherapy in LC. Fourth, we analyzed correlation and interaction between m6A-related genes and tumor drug resistance, which could provide a new therapeutic choice to decrease drug resistance in LC. However, there are still several restrictions. First, due to limited statistics, optimized gene-signature model should be concerned in a large-scale clinical cohort study. Second, more in vivo or vitro experiments should be performed for further validation. Third, our study employed only m6A-relaataed genes to establish risk prognostic signature, which may exclude some hub biomarkers.

## Conclusion

In conclusion, our study comprehensively analyzed expression, prognosis, TME, and drug sensitivity of m6A-related genes in LUAD and LUSC. The gene signature consisting of ZC3H13, CBLL1, ELAVL1 and YTHDF1, were validated with different analyses and from GEO dataset. In short, our study provided novel marker for predicting the prognostic value as well as developed a novel direct based on m6A-related genes for reversing tumor progression in LCs.

## Supplementary Information

**Additional file 1**. The workflow and study design of the analysis steps.

**Additional file 2**. Summary of the LC mutation information and the 10 DEMGs mutation information as well as CNV analysis of m6A-related genes in LC (GSCALite).

**Additional file 3**. The cluster of LC cancer based on m6A-related genes.

**Additional file 4**.The expression level of immune checkpoints in high-risk and low-risk groups.

**Additional file 5**. Infiltration of gene expression, overall survival, mutation profile and the comparison of tumor infiltration levels in m6A-related genes with different somatic copy number alterations for LUAD and LUSC in TIMER2.0.

**Additional file 6**. Infiltration of gene expression, overall survival, mutation profile and the comparison of tumor infiltration levels in m6A-related genes with different somatic copy number alterations for LUAD and LUSC in TIMER2.0.

**Additional file 7**. Infiltration of gene expression, overall survival, mutation profile and the comparison of tumor infiltration levels in m6A-related genes with different somatic copy number alterations for LUAD and LUSC in TIMER2.0.

**Additional file 8**. The heatmap of correlation between the immune characteristics and the four hub DEMGs in LUAD and LUSC from TISID.

**Additional file 9**. Heatmap of DNA methylation expression levels of DEMGs in LUAD and LUSC by MethSurv platform.

**Additional file 10**. Overall survival analysis of HPA and validation of gene expressions of 4 hub DEMGs from GSCALite.

**Additional file 11**. Summary of Gene-CpG in LC

## Data Availability

Publicly available data sets were analyzed in this study. The data can be found below: 1. TCGA, https://www.cancer.gov/, 2. GEO, https://www.ncbi.nlm.nih.gov/geo/.

## Abbreviations

LC: Lung cancer
LUAD: Lung adenocarcinoma
LUSC: Lung squamous cell carcinoma
m6A: N6-methyladenosine
CBLL1: 1 M6A-related protein gene
GO: Gene ontology
PPI: Protein–protein interaction
CNV: Copy number variations
PAC: Proportion of ambiguous clustering
PCA: Principal component analysis
ROC: Receiver operating characteristic curve
TME: Tumor microenvironment
sCNAs: Somatic copy number alterations
RNA-Seq: Quantitative transcriptomics data
GTEx: Genotype tissue expression
CDF: Cumulative distribution function
HCC: Hepatocellular carcinoma
CRC: Colorectal cancer
CCND1: Cyclin D1
CDK2: Cyclin-dependent kinase 2
CDK4: Cyclin-dependent kinase 4
ELAVL1/HuR: Embryonic lethal abnormal vision like 1/human antigen R
ERBB2: Epidermal growth factor receptor-2
COX-2: Cyclooxygenase-2
APC: Antigen presenting cell
TKI: Tyrosine kinase inhibitors
EMT: Epithelial to mesenchymal transition
SCLC: Small cell lung cancer

## References

[1] Sung H (2021). Global cancer statistics 2020: GLOBOCAN estimates of incidence and mortality worldwide for 36 cancers in 185 countries. CA Cancer J Clin.

[2] Howlader N (2020). The effect of advances in lung-cancer treatment on population mortality. N Engl J Med.

[3] Bade BC, Dela Cruz CS (2020). Lung cancer 2020: epidemiology, etiology, and prevention. Clin Chest Med.

[4] Cowper PA (2021). Initial and longitudinal cost of surgical resection for lung cancer. Ann Thorac Surg.

[5] Doroshow DB (2019). Immunotherapy in non-small cell lung cancer: facts and hopes. Clin Cancer Res.

[6] Imyanitov EN, Iyevleva AG, Levchenko EV (2021). Molecular testing and targeted therapy for non-small cell lung cancer: current status and perspectives. Crit Rev Oncol Hematol.

[7] Oudkerk M (2021). Lung cancer LDCT screening and mortality reduction—evidence, pitfalls and future perspectives. Nat Rev Clin Oncol.

[8] Vinod SK, Hau E (2020). Radiotherapy treatment for lung cancer: current status and future directions. Respirology (Carlton, VIC).

[9] Arbour KC, Riely GJ (2019). Systemic therapy for locally advanced and metastatic non-small cell lung cancer: a review. JAMA.

[10] Seijo LM (2019). Biomarkers in lung cancer screening: achievements, promises, and challenges. J Thorac Oncol.

[11] Lan T (2019). KIAA1429 contributes to liver cancer progression through N6-methyladenosine-dependent post-transcriptional modification of GATA3. Mol Cancer.

[12] Song H (2019). METTL3 and ALKBH5 oppositely regulate m(6)A modification of TFEB mRNA, which dictates the fate of hypoxia/reoxygenation-treated cardiomyocytes. Autophagy.

[13] He PC, He C (2021). m(6) A RNA methylation: from mechanisms to therapeutic potential. EMBO J.

[14] Zhao W (2020). Epigenetic regulation of m(6)A modifications in human cancer. Mol Ther Nucleic acids.

[15] Huang X (2020). m6A RNA methylation regulators could contribute to the occurrence of chronic obstructive pulmonary disease. J Cell Mol Med.

[16] Wiener D, Schwartz S (2021). The epitranscriptome beyond m(6)A. Nat Rev Genet.

[17] He L (2019). Functions of N6-methyladenosine and its role in cancer. Mol Cancer.

[18] Jiang X (2021). The role of m6A modification in the biological functions and diseases. Signal Transduct Target Ther.

[19] Wei J, He C (2019). Site-specific m(6)A editing. Nat Chem Biol.

[20] Mayakonda A (2018). Maftools: efficient and comprehensive analysis of somatic variants in cancer. Genome Res.

[21] Liu CJ (2018). GSCALite: a web server for gene set cancer analysis. Bioinformatics (Oxford, England).

[22] Vaulet T (2021). Data-driven derivation and validation of novel phenotypes for acute kidney transplant rejection using semi-supervised clustering. J Am Soc Nephrol.

[23] Wilkerson MD, Hayes DN (2010). ConsensusClusterPlus: a class discovery tool with confidence assessments and item tracking. Bioinformatics (Oxford, England).

[24] Sauerbrei W, Royston P, Binder H (2007). Selection of important variables and determination of functional form for continuous predictors in multivariable model building. Stat Med.

[25] Hu X (2017). Multigene signature for predicting prognosis of patients with 1p19q co-deletion diffuse glioma. Neuro Oncol.

[26] Zhou Z (2018). Identification of an energy metabolism-related signature associated with clinical prognosis in diffuse glioma. Aging.

[27] Hinshaw DC, Shevde LA (2019). The tumor microenvironment innately modulates cancer progression. Can Res.

[28] Beibei Ru (2019). TISIDB: an integrated repository portal for tumor-immune system interactions. Bioinformatics.

[29] Li T (2017). TIMER: a web server for comprehensive analysis of tumor-infiltrating immune cells. Can Res.

[30] Li T (2020). TIMER2.0 for analysis of tumor-infiltrating immune cells. Nucleic Acids Res.

[31] Pontén F, Jirström K, Uhlen M (2008). The Human Protein Atlas—a tool for pathology. J Pathol.

[32] Vijayachitra M (2018). MethSurv: a web tool to perform multivariable survival analysis using DNA methylation data. Epigenomics.

[33] Bai Y (2019). YTHDF1 regulates tumorigenicity and cancer stem cell-like activity in human colorectal carcinoma. Front Oncol.

[34] Shi Y (2019). YTHDF1 links hypoxia adaptation and non-small cell lung cancer progression. Nat Commun.

[35] Han D (2019). Anti-tumour immunity controlled through mRNA m(6)A methylation and YTHDF1 in dendritic cells. Nature.

[36] Jin D (2020). m(6)A demethylase ALKBH5 inhibits tumor growth and metastasis by reducing YTHDFs-mediated YAP expression and inhibiting miR-107/LATS2-mediated YAP activity in NSCLC. Mol Cancer.

[37] Sheng H (2020). YTH domain family 2 promotes lung cancer cell growth by facilitating 6-phosphogluconate dehydrogenase mRNA translation. Carcinogenesis.

[38] Zhu D (2019). ZC3H13 suppresses colorectal cancer proliferation and invasion via inactivating Ras-ERK signaling. J Cell Physiol.

[39] Gong PJ (2020). Analysis of N6-methyladenosine methyltransferase reveals METTL14 and ZC3H13 as tumor suppressor genes in breast cancer. Front Oncol.

[40] Hui L (2019). CBLL1 is highly expressed in non-small cell lung cancer and promotes cell proliferation and invasion. Thorac Cancer.

[41] Qiu HB (2019). Downregulation of long non-coding RNA XIST inhibits cell proliferation, migration, invasion and EMT by regulating miR-212-3p/CBLL1 axis in non-small cell lung cancer cells. Eur Rev Med Pharmacol Sci.

[42] Pabis M (2019). HuR biological function involves RRM3-mediated dimerization and RNA binding by all three RRMs. Nucleic Acids Res.

[43] Wang ZY, Yin L (2015). Estrogen receptor alpha-36 (ER-α36): A new player in human breast cancer. Mol Cell Endocrinol.

[44] Dong R (2007). Stabilization of Snail by HuR in the process of hydrogen peroxide induced cell migration. Biochem Biophys Res Commun.

[45] Zhou Y (2016). Loss of scribble promotes snail translation through translocation of HuR and enhances cancer drug resistance. J Biol Chem.

[46] Cheng T, Zhan X (2017). Pattern recognition for predictive, preventive, and personalized medicine in cancer. EPMA J.

[47] Brandes M, Willimann K, Moser B (2005). Professional antigen-presentation function by human gammadelta T Cells. Science (New York, NY).

[48] Liu Z (2008). Protective immunosurveillance and therapeutic antitumor activity of gammadelta T cells demonstrated in a mouse model of prostate cancer. J Immunol (Baltimore, Md: 1950).

[49] Helmink BA (2020). B cells and tertiary lymphoid structures promote immunotherapy response. Nature.

[50] Bocchino M (2021). Dendritic cells are the intriguing players in the puzzle of idiopathic pulmonary fibrosis pathogenesis. Front Immunol.

[51] Monzó M (1999). Paclitaxel resistance in non-small-cell lung cancer associated with beta-tubulin gene mutations. J Clin Oncol.

[52] Blagosklonny MV (1996). Taxol-induced apoptosis and phosphorylation of Bcl-2 protein involves c-Raf-1 and represents a novel c-Raf-1 signal transduction pathway. Can Res.

[53] Shi Y (2005). Optimal classes of chemotherapeutic agents sensitized by specific small-molecule inhibitors of akt in vitro and in vivo. Neoplasia (New York, NY).

[54] Baudin E (2019). Unmet medical needs in pulmonary neuroendocrine (carcinoid) neoplasms. Neuroendocrinology.

[55] Wolin EM (2017). Advances in the diagnosis and management of well-differentiated and intermediate-differentiated neuroendocrine tumors of the lung. Chest.

[56] Ding N (2020). Chidamide increases the sensitivity of non-small cell lung cancer to crizotinib by decreasing c-MET mRNA methylation. Int J Biol Sci.

[57] Meng Q (2020). Dissecting the m(6)A methylation affection on afatinib resistance in non-small cell lung cancer. Pharmacogenomics J.

[58] Demuth C (2018). The T790M resistance mutation in EGFR is only found in cfDNA from erlotinib-treated NSCLC patients that harbored an activating EGFR mutation before treatment. BMC Cancer.

[59] Yun CH (2008). The T790M mutation in EGFR kinase causes drug resistance by increasing the affinity for ATP. Proc Natl Acad Sci USA.

[60] Tetsu O (2016). Drug resistance to EGFR inhibitors in lung cancer. Chemotherapy.

[61] Jakobsen KR (2017). MET amplification and epithelial-to-mesenchymal transition exist as parallel resistance mechanisms in erlotinib-resistant, EGFR-mutated, NSCLC HCC827 cells. Oncogenesis.

[62] Lian T, Li C, Wang H (2019). Trametinib in the treatment of multiple malignancies harboring MEK1 mutations. Cancer Treat Rev.

[63] Wang X (2016). N1-guanyl-1,7-diaminoheptane enhances the chemosensitivity of NSCLC cells to cetuximab through inhibition of eukaryotic translation initiation factor 5A2 activation. Eur Rev Med Pharmacol Sci.

