# Supplementary figures and images for: Study on the prognosis, immune and drug resistance of m6A-related genes in lung cancer
Source: BMC Bioinformatics. 2022 Oct 19;23:437. doi: 10.1186/s12859-022-04984-5 (PMC9583491; doi:10.1186/s12859-022-04984-5)

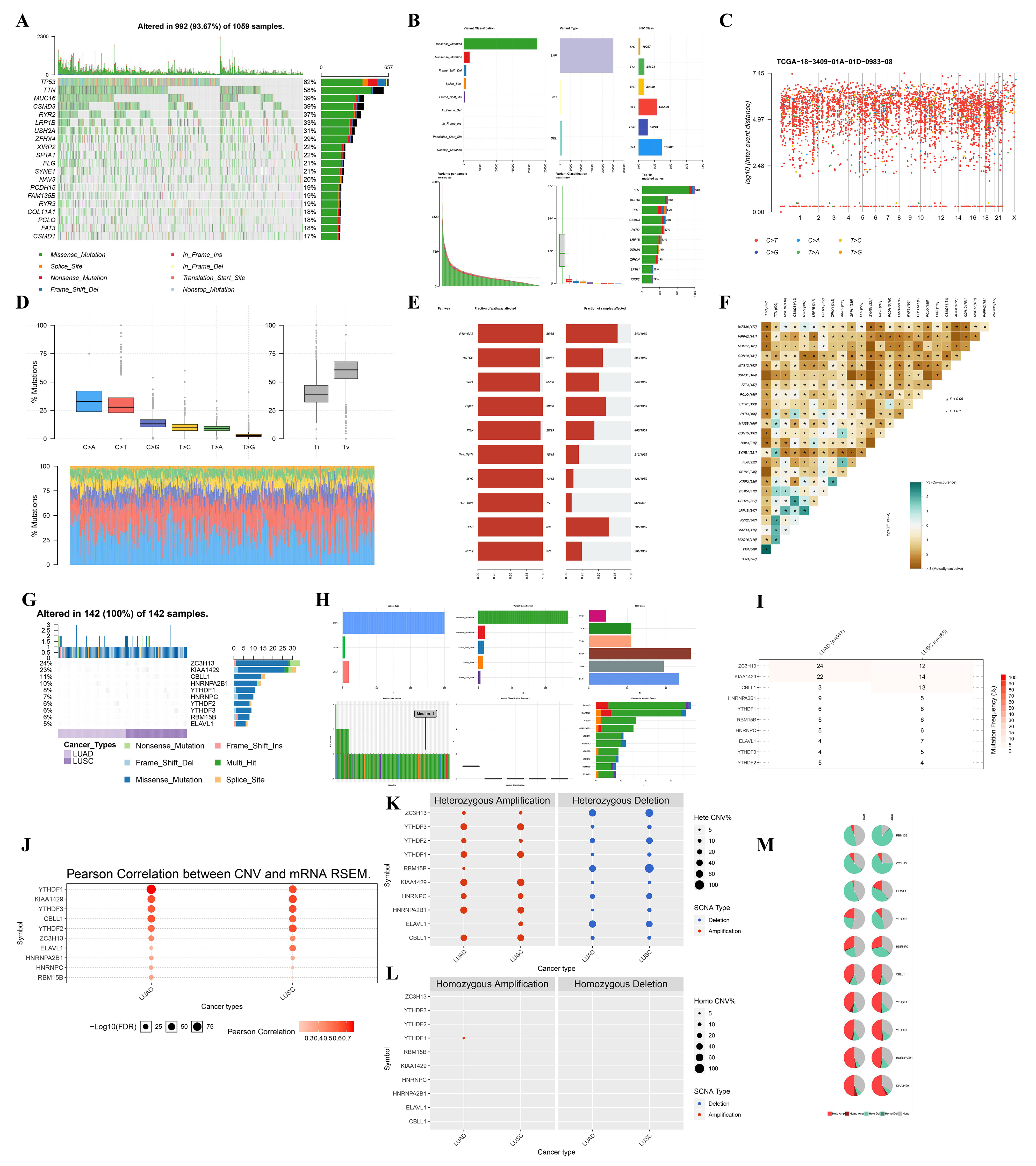

Supplement: Supplementary file 2 — Additional file 2. Summary of the LC mutation information and the 10 DEMGs mutation information as well as CNV analysis of m6A-related genes in LC (GSCALite). [file 12859_2022_4984_MOESM2_ESM.jpg]

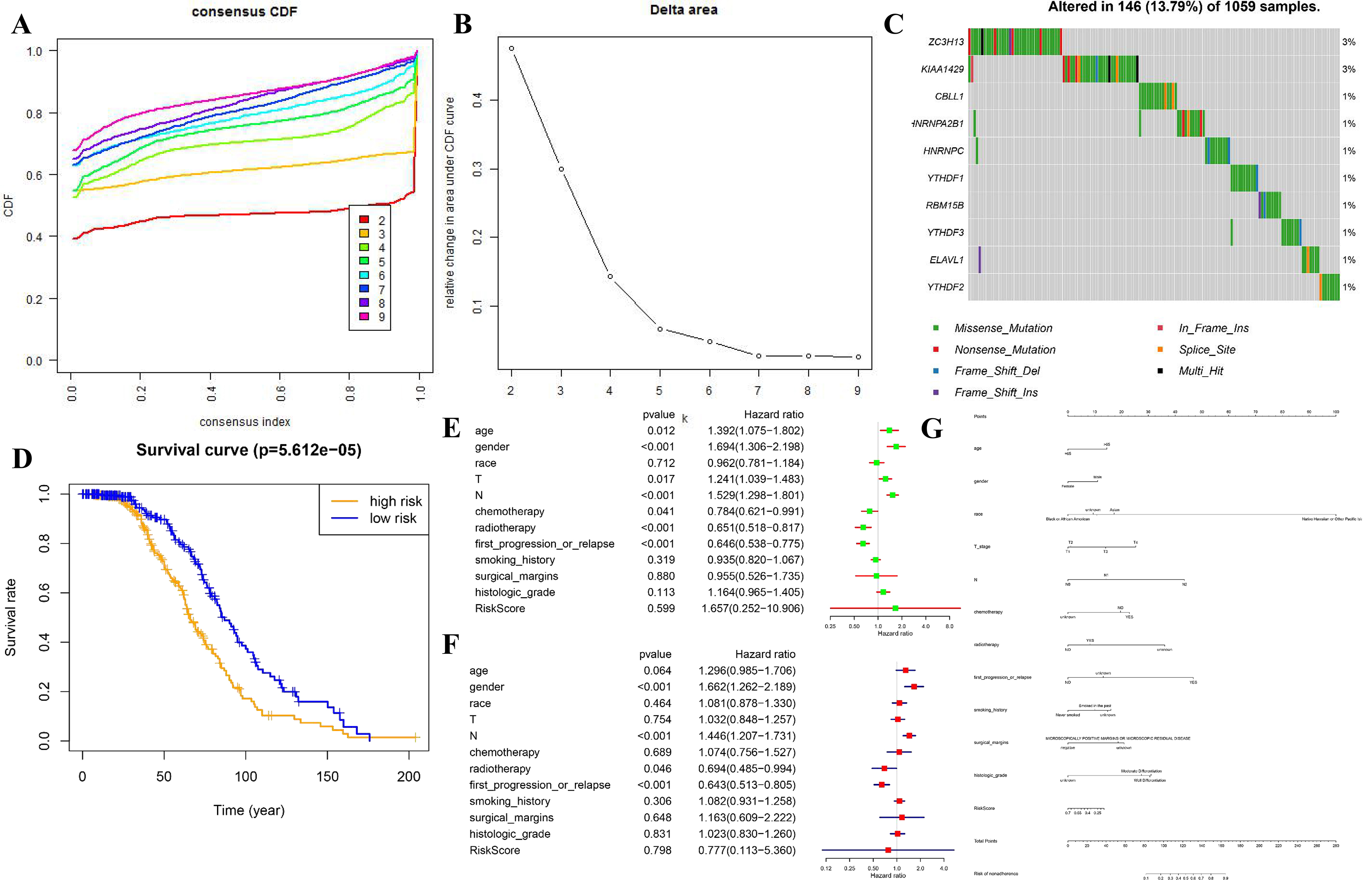

Supplement: Supplementary file 3 — Additional file 3. The cluster of LC cancer based on m6A-related genes. [file 12859_2022_4984_MOESM3_ESM.jpg]

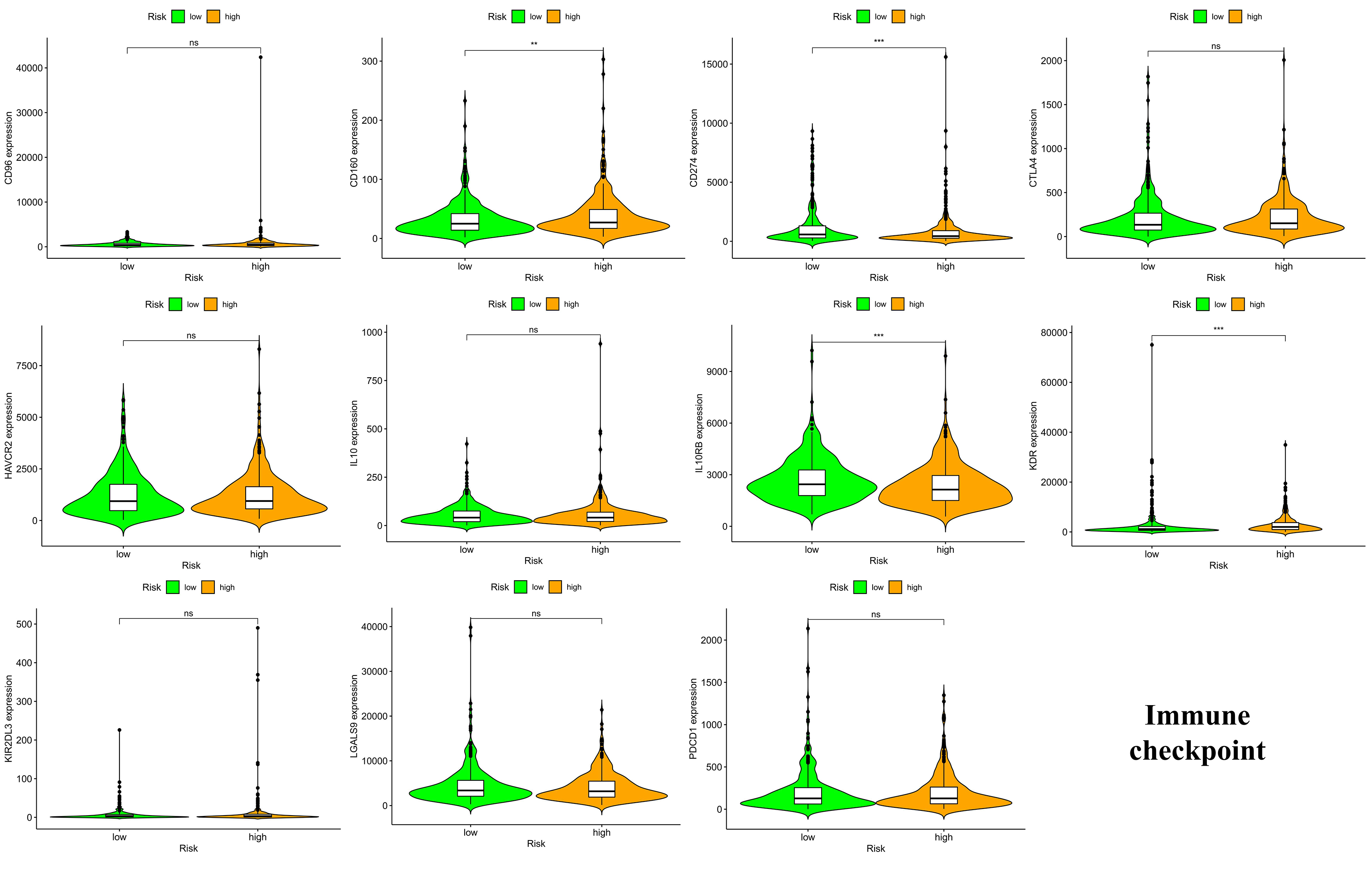

Supplement: Supplementary file 4 — Additional file 4.The expression level of immune checkpoints in high-risk and low-risk groups. [file 12859_2022_4984_MOESM4_ESM.jpg]

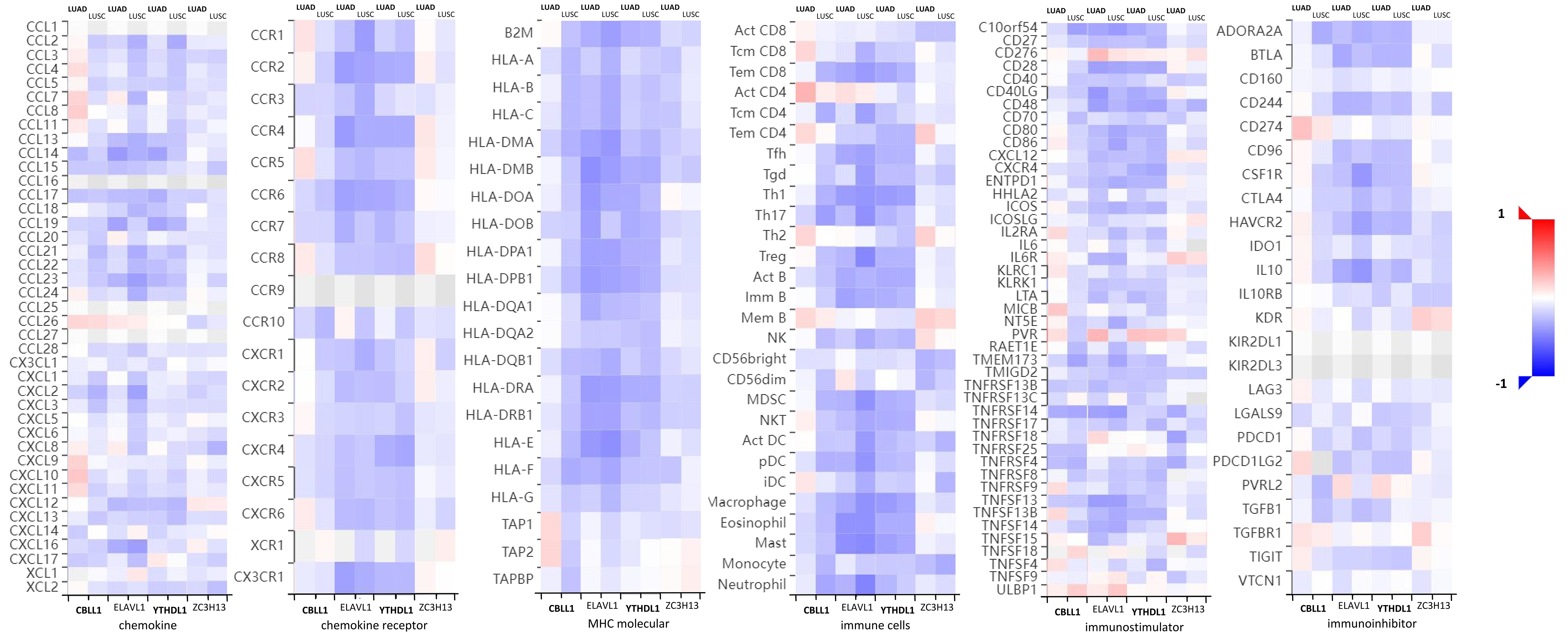

Supplement: Supplementary file 8 — Additional file 8. The heatmap of correlation between the immune characteristics and the four hub DEMGs in LUAD and LUSC from TISID. [file 12859_2022_4984_MOESM8_ESM.jpg]
