# Supplementary material for: Study on the prognosis, immune and drug resistance of m6A-related genes in lung cancer
Source: BMC Bioinformatics. 2022 Oct 19;23:437. doi: 10.1186/s12859-022-04984-5 (PMC9583491; doi:10.1186/s12859-022-04984-5)
Supplement: Supplementary file 11 — Additional file 11. Summary of Gene-CpG in LC [file 12859_2022_4984_MOESM11_ESM.docx]

| **Gene-CpG** | **Cancer** | **HR** | **LR_test_p-value** |
| --- | --- | --- | --- |
| CBLL1-Body;3'UTR-Open_Sea-cg17158101 | LUAD | 0.888 | 0.52 |
| CBLL1-Body;3'UTR-Open_Sea-cg17158101 | LUSC | 1.106 | 0.54 |
| CBLL1-Body-S_Shelf-cg27396403 | LUAD | 1.201 | 0.26 |
| CBLL1-Body-S_Shelf-cg27396403 | LUSC | 1.208 | 0.28 |
| CBLL1-Body-Open_Sea-cg19318403 | LUAD | 0.889 | 0.46 |
| ELAVL1-Body-Island-cg00879606 | LUAD | 0.884 | 0.50 |
| ELAVL1-Body-Island-cg00879606 | LUSC | 1.136 | 0.43 |
| ELAVL1-Body-S_Shore-cg01043729 | LUAD | 0.697 | 0.0418 * |
| ELAVL1-Body-S_Shore-cg01043729 | LUSC | 0.799 | 0.22 |
| ELAVL1-Body-Island-cg09689465 | LUAD | 1.113 | 0.57 |
| ELAVL1-Body-Island-cg09689465 | LUSC | 0.837 | 0.27 |
| ELAVL1-Body-Island-cg26278134 | LUAD | 1.368 | 0.05 |
| ELAVL1-Body-Island-cg26278134 | LUSC | 0.747 | 0.07 |
| ELAVL1-3'UTR-N_Shelf-cg03355359 | LUAD | 1.41 | 0.07 |
| ELAVL1-3'UTR-N_Shelf-cg03355359 | LUSC | 1.157 | 0.37 |
| ELAVL1-Body-S_Shelf-cg23254334 | LUAD | 1.194 | 0.36 |
| ELAVL1-Body-S_Shelf-cg23254334 | LUSC | 1.46 | 0.06 |
| HNRNPA2B1-3'UTR-Open_Sea-cg23401436 | LUAD | 0.813 | 0.24 |
| HNRNPA2B1-3'UTR-Open_Sea-cg23401436 | LUSC | 1.364 | 0.06 |
| HNRNPC-5'UTR-Open_Sea-cg15413271 | LUAD | 1.251 | 0.16 |
| HNRNPC-5'UTR-Open_Sea-cg15413271 | LUSC | 0.816 | 0.27 |
| HNRNPC-TSS1500-S_Shore-cg13673563 | LUAD | 0.817 | 0.21 |
| HNRNPC-TSS1500-S_Shore-cg13673563 | LUSC | 1.129 | 0.53 |
| HNRNPC-5'UTR-Open_Sea-cg17002899 | LUAD | 0.758 | 0.08 |
| KIAA1429-Body-N_Shelf-cg00161683 | LUAD | 1.364 | 0.10 |
| KIAA1429-Body-N_Shelf-cg00161683 | LUSC | 0.747 | 0.07 |
| RBM15B-1stExon-Island-cg03037622 | LUAD | 1.161 | 0.35 |
| RBM15B-1stExon-Island-cg03037622 | LUSC | 1.127 | 0.57 |
| RBM15B-1stExon-Island-cg15035459 | LUAD | 1.072 | 0.67 |
| RBM15B-1stExon-Island-cg15035459 | LUSC | 1.155 | 0.49 |
| RBM15B-1stExon-N_Shore-cg07620844 | LUAD | 0.81 | 0.24 |
| RBM15B-1stExon-Island-cg27215641 | LUAD | 1.34 | 0.07 |
| YTHDF1-Body-N_Shelf-cg10201192 | LUAD | 0.882 | 0.51 |
| YTHDF1-Body-N_Shelf-cg10201192 | LUSC | 1.396 | 0.08 |
| YTHDF1-3'UTR-Open_Sea-cg25041371 | LUAD | 1.045 | 0.79 |
| YTHDF1-3'UTR-Open_Sea-cg25041371 | LUSC | 1.313 | 0.13 |
| YTHDF2-Body-Open_Sea-cg07908197 | LUAD | 0.805 | 0.24 |
| YTHDF2-Body-Open_Sea-cg07908197 | LUSC | 0.847 | 0.37 |
| YTHDF3-Body-S_Shelf-cg03922048 | LUAD | 0.861 | 0.40 |
| YTHDF3-Body-S_Shelf-cg03922048 | LUSC | 1.071 | 0.69 |
| ZC3H13-Body-Open_Sea-cg08531917 | LUAD | 0.776 | 0.12 |
| ZC3H13-Body-Open_Sea-cg08531917 | LUSC | 1.196 | 0.33 |
| ZC3H13-Body-Open_Sea-cg17598724 | LUAD | 1.173 | 0.33 |
| ZC3H13-Body-Open_Sea-cg17598724 | LUSC | 1.248 | 0.21 |
| ZC3H13-Body-Open_Sea-cg02122052 | LUAD | 0.829 | 0.30 |
| ZC3H13-Body-Open_Sea-cg02122052 | LUSC | 1.249 | 0.17 |
| ZC3H13-Body-Open_Sea-cg06720244 | LUAD | 0.718 | 0.0401 * |
| ZC3H13-Body-Open_Sea-cg06720244 | LUSC | 1.234 | 0.20 |
| ZC3H13-Body-Open_Sea-cg22152521 | LUAD | 0.77 | 0.10 |
| ZC3H13-Body-Open_Sea-cg05724742 | LUAD | 0.751 | 0.11 |
| ZC3H13-Body-Open_Sea-cg09598590 | LUAD | 0.887 | 0.45 |
| ZC3H13-Body-Open_Sea-cg20891306 | LUAD | 0.755 | 0.08 |

**Supplementary Table1:** Prognostic Value of Single CpG of DEMGs in LUAD and LUSC by MethSurv platform. The threshold of significance was LR Test p-value <0.05. ELAVL1 and ZC3H13 expressed significantly different between low and high risk groups for LUAD.
